# Proteobacteria with chemosynthetic potential are highly prevalent in the gills of *Hypoplectrus* reef fishes

**DOI:** 10.1371/journal.pgen.1012266

**Published:** 2026-08-28

**Authors:** Sabrin Abdelghany, Martin Helmkampf, Matthew S. Schechter, Iva A. Veseli, Matthieu Leray, A. Murat Eren, Oscar Puebla

**Affiliations:** 1 Leibniz Centre for Tropical Marine Research (ZMT), Bremen, Germany; 2 Institute for Chemistry and Biology of the Marine Environment (ICBM), School of Mathematics and Science, Carl von Ossietzky Universität Oldenburg, Oldenburg, Germany; 3 National Institute of Oceanography and Fisheries (NIOF), Cairo, Egypt; 4 Committee on Microbiology, University of Chicago, Chicago, Illinois, United States of America; 5 Helmholtz Institute for Functional Marine Biodiversity at Oldenburg, Oldenburg, Germany; 6 School of Biological Sciences, Swire Institute of Marine Science, The University of Hong Kong, Hong Kong, SAR, China; 7 Alfred Wegener Institute, Helmholtz Centre for Polar and Marine Research, Bremerhaven, Germany; 8 Max Planck Institute for Marine Microbiology, Bremen, Germany; 9 Smithsonian Tropical Research Institute (STRI), Panama City, Panama, Republic of Panama; Kiel University: Christian-Albrechts-Universitat zu Kiel, GERMANY

## Abstract

Fishes host a diverse microbiome in their gills, but a broad characterization of this microbiome at the metagenomic level is lacking. Here, we apply genome-resolved metagenomics to the gills of the hamlets (*Hypoplectrus* spp), a group of reef fishes from the Greater Caribbean. The analysis of 353 gill samples from 15 hamlet species collected at eight locations over 13 years revealed a stark contrast between the gill microbiota and reef water microbial communities, indicating a distinct and specific gill microbiome. A total of 70 gill-associated metagenome-assembled genomes (MAGs) were recovered. These MAGs belong to 17 lineages, most of which are novel. They relate to known fish gill pathogens, fish gut microbes, free-living and biofilm-associated taxa, indicating that the gill microbiome was assembled from a collection of distinct eco-evolutionary trajectories. The MAGs harbor diverse metabolic modules, involved notably in nitrogen cycling, antibiotic production and biofilm formation, revealing a highly dynamic microbial ecosystem. One lineage in the Burkholderiaceae family was outstandingly prevalent across fish host species, sampling locations and years. Its genome encoded complete metabolic modules for carbon fixation and sulfur oxidation, indicating chemosynthetic potential. To the best of our knowledge, this is the first line of evidence that fishes may host sulfur-oxidizing chemosynthetic bacteria in their gills. The functional significance of this chemosynthetic potential for the fish host or other members of the gill microbiome remains to be established. The high prevalence of this lineage allowed to build a pangenome. It revealed large-scale geographic structure (western Caribbean, eastern Caribbean and Gulf of Mexico), which parallels the phylogenomic pattern observed in the hamlets. Overall, our findings point to complex fish host-microbe and microbe-microbe eco-evolutionary interactions in the gills that may influence fish physiology, homeostasis and immune response.

## Author summary

The ability of some bacteria to fix carbon dioxide and produce energy through the oxidation of inorganic compounds was discovered more than a century ago. This biological process, now referred to as chemolithoautotrophy, is a form of chemosynthesis—as opposed to photosynthesis whereby energy is obtained from the sun. Chemosynthetic bacteria have been documented in a variety of marine environments and invertebrate hosts, with whom they can form symbiotic associations, but this is poorly documented in fishes. Here, we analyze an extensive dataset of 44 billion DNA sequences from gills of a group of Caribbean reef fishes. We show that one novel bacterial lineage is outstandingly prevalent across fish host species, locations and sampling years, and that its genome encodes all the genes required for chemosynthesis. To the best of our knowledge, this constitutes the first line of evidence that fishes may host sulfur-oxidizing chemosynthetic bacteria in their gills. The functional significance of this chemosynthetic potential for the fish host or other gill microbes remains to be established.

## Introduction

The fish gills constitute the major interface between the body and its environment through which gas exchange occurs. They also contribute to eliminate nitrogenous waste [1,2] and maintain homeostasis through the regulation of osmotic pressure, pH and temperature in some cases [2–4]. Furthermore, the gills constitute a mucosal barrier that contributes to defense against pathogens and maintenance of the fish immune response [5,6]. All these functions are critical for the physiological adaptability of fishes to rapidly changing natural habitats and to aquaculture environments.

Fish gills also harbor a microbiome [7–9]. Through its interactions with mucosal immunity [10] and its potential contribution to ammonia detoxification [11,12], the gill microbiome may play an important role in fish physiology and health. Metabarcoding of 16S ribosomal RNA (rRNA) indicates that the fish gill microbiome is diverse and dominated by the bacterial phyla Pseudomonadota (Proteobacteria), Actinomycetota (Actinobacteria), Bacteroidota (Bacteroidetes) and Bacillota (Firmicutes) [7–9,13–15]. Nevertheless, while some fish gill-associated bacteria have been successfully cultivated and characterized [16,17], the vast majority of microorganisms that are detected in metabarcoding surveys are missing in culture and their functional role is poorly understood [18,19]. By enabling the recovery of microbial genomes directly from environmental samples [20], shotgun metagenomics yields new opportunities to characterize the genomes of gill-associated bacteria without cultivation, offering an improved understanding of the fish gill microbiome [21]. Yet a large fraction of studies on the fish microbiome focus on the gut [15], and the studies that target fish gills with shotgun metagenomics tend to focus on specific pathogens [22–24]. A broad metagenomic characterization of fish gill microbiome, i.e., one that does not target any member of the gill microbiome in particular, is lacking.

**Fig 1:**
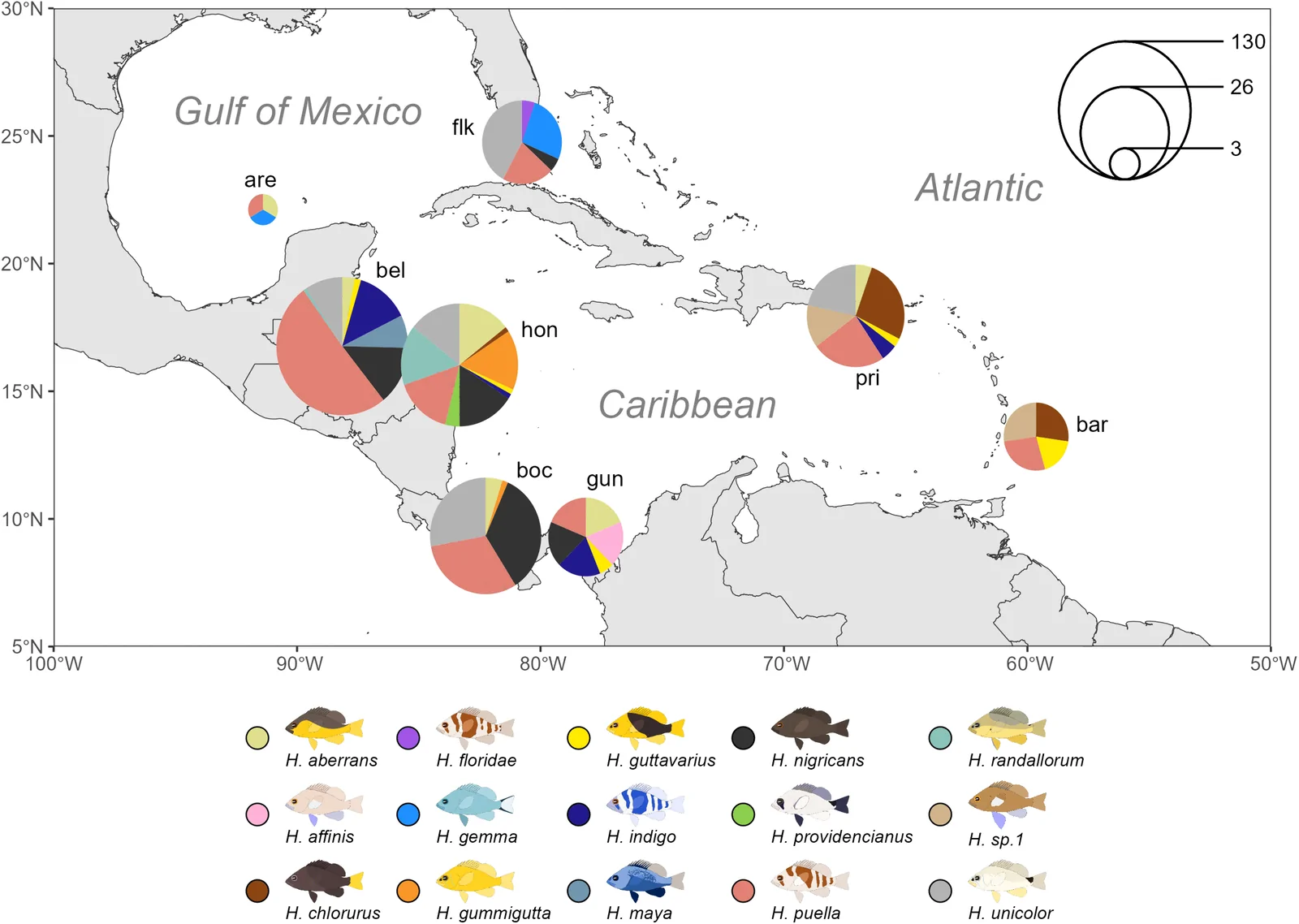
Sampling design. The dataset consisted of 353 shotgun-sequenced gill tissue samples from 15 hamlet species collected at eight Greater Caribbean locations: Cayo Arenas (are) and the Florida Keys (flk) in the Gulf of Mexico; Belize (bel), Honduras (hon), Bocas del Toro (boc) and Guna Yala (gun) in the Western Caribbean; and Puerto Rico (pri) and Barbados (bar) in the Eastern Caribbean. Additionally, seven gill samples of *Serranus* were analyzed – three samples of *S. tortugarum* and two samples of *S. tabacarius* and *S. tigrinus*, collected in Honduras and Bocas del Toro. Basemap from the R package rnaturalearth (Natural Earth Admin 0 – Countries dataset, large scale, https://www.naturalearthdata.com).

Here, we fill this knowledge gap by broadly applying genome-resolved metagenomics to the gills of the hamlets (*Hypoplectrus* spp). Hamlets are a group of fishes found throughout the Greater Caribbean, primarily associated with coral reefs and feeding on small fishes and invertebrates. The group includes 19 + species [25,26] that are ecologically very similar [27,28], highly sympatric (up to nine species on a single reef), and differ almost exclusively in terms of color pattern and mate choice. They are extremely close genetically ($F_{ST}$ between pairs of sympatric species 0.001-0.191) and form a very shallow evolutionary radiation that appears to be exceptionally recent and rapid, making them a rare model system for the study of speciation and radiation in the marine environment [29–33]. In this context, we shotgun-sequenced 353 hamlet gill tissue samples from 15 species and eight locations collected over 13 years (Fig 1). This dataset was used to study the fish host genome [32–36] but also provides the opportunity to characterize the gill microbiome of the radiation at the metagenomic level, which is what we do here. Considering the high genetic and ecological similarity of hamlet species, we do not expect important differences among species in terms of their gill microbiome. Given how little is known about the fish gill microbiome from a metagenomic and functional perspective, we adopt an exploratory approach as opposed to a hypothesis-driven one.

## Results and discussion

### The gill microbiome is diverse and conserved across species, locations and years

We started by characterizing the taxonomic composition of the gill microbiome at the phylum, class, order and family levels using a genomic read classification approach (S2-S5 Figs). A total of 1.9 billion quality-filtered reads were retained after removing fish host sequences, representing 4.3% of the original dataset (43.8 billion reads). Of these, 1.4 billion reads (3.2% of the original dataset) were retained after further filtering out reads classified as metazoans, photosynthetic eukaryotes (Viridiplantae, Rhodophyta, Haptophyta and Bacillariophyta) and PhiX. In agreement with fish gill metabarcoding studies [7–9,13–15], the results indicated a high prevalence of Pseudomonadota (particularly the classes Gammaproteobacteria and Betaproteobacteria), Actinomycetota, Bacteroidota and Bacillota in the gills. Nevertheless, the composition of the hamlet gill microbiome differed from other marine and reef fishes at low taxonomic levels (order and below). For example, a high prevalence of *Shewanella*—a genus of Gammaproteobacteria in the order Alteromonadales—was reported in other tropical marine fishes [8,9,14] but this genus was not detected and Alteromonadales was not identified as a dominant order by our read classification analysis. This result is in line with evidence that the microbiome of reef fishes is partitioned among species [37,38]. Furthermore, we identified protists, fungi, archaea and viruses in all samples, providing a taxonomically broader view of the fish gill microbiome diversity than metabarcoding and indicating that these phyla are an integral part of the gill microbiome (S2-S4 Figs).

Marginal Permutational Multivariate Analysis of Variance (PERMANOVA) indicated that the composition of the gill microbiome is significantly structured with respect to sequencing batch and fish host species, but that these factors explain only 7.0% and 2.6% of variation, respectively (S1 Table). This was confirmed by Principal Coordinates Analysis (PCoA), which showed that the gill microbiome community composition is largely shared across fish host species, locations and years (S6 Fig). These results validate our hypothesis that the gill microbiome is largely shared among hamlet species, which is line with their high ecological and genetic similarity. We found no significant interactions between fish host species and either sampling location or sampling year after accounting for sequencing batch effects, indicating that the small but significant fish host species effect is not location- or year-specific.

A few fish host species/year/location samples presented an atypical gill microbiome composition, but these often had a low sample size. For example, *H. nigricans* from the Florida Keys (2017) is characterized by 35.5% Bacteroidaceae relative read abundance (as opposed to <8.5% in other samples) and *H. guttavarius* from Honduras (2006) by 46.9% Bartonellaceae relative read abundance (as opposed to <12.8% in other samples, S5 Fig), but these two samples are represented by just one fish host individual. These differences in gill microbiome composition are therefore likely to reflect in large part individual variation as opposed to fish host species/year/location variation.

### The gill microbiome is distinct from reef seawater

In order to evaluate to what extent the gill microbiome differs from the reef water microbiome, we also analyzed shotgun sequences from reef water collected at one of our sampling location (Bocas del Toro, Panama) [39,40]. For this we used the EcoPhylo workflow [41], a novel approach that profiles the distribution of gene family homologs across metagenomic samples, considering the ribosomal protein single-copy core gene *rpL16* as a proxy for the presence of microbial populations.

**Fig 2:**
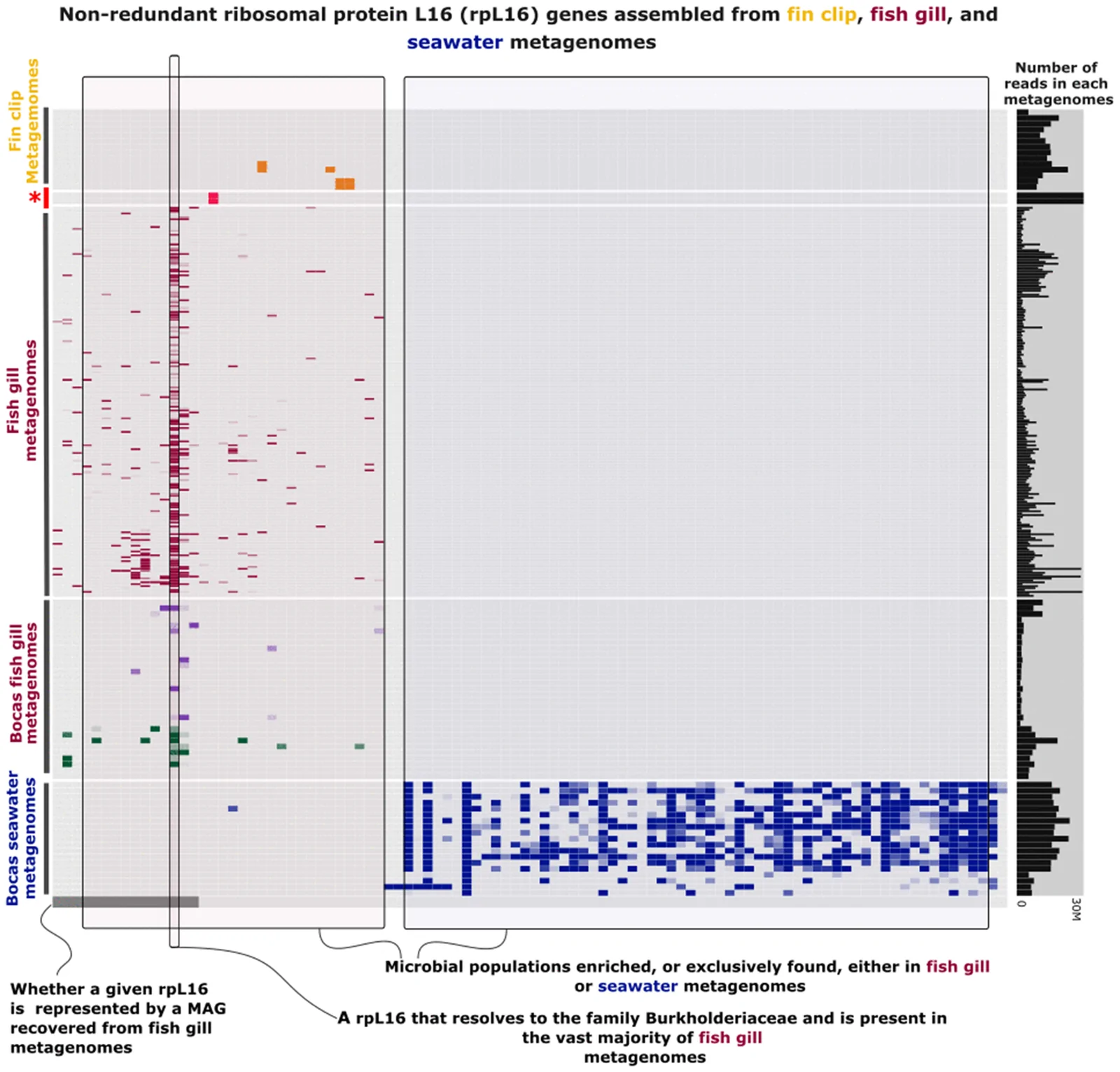
Microbiome community composition in the gills and seawater based on the *rpL16* gene family. The heatmap shows the detection (70-100%) of the different *rpL16* clades (columns) in the gills versus seawater metagenomes (rows), indicating little overlap in community composition between the gill and seawater microbiomes with just one clade out of 94 detected in both environments. Note the high prevalence of one clade in the gills (and conversely its absence in seawater), which corresponds to a novel lineage in the Burkholderiaceae family. Another striking pattern is the lower microbial diversity in the gills compared to seawater, indicating a more specialized microbiome in the gills. The grey part of the horizontal bar at the bottom represents the *rpL16* clades for which a MAG was assembled, indicating that despite the large sampling and sequencing effort, the bacterial lineages that were recovered as MAGs from the gills represent just 54% of the lineages detected by the *rpL16* marker in the gills. Blue: seawater metagenomes from Bocas del Toro (2017). Green: hamlet gill metagenomes from Bocas del Toro (2017). Purple: hamlet gill metagenomes from Bocas del Toro (2004-2005). Crimson: hamlet gill metagenomes from other locations and years. Red: *Serranus spp* gill metagenomes. Yellow: hamlet fin clip metagenomes. The number of reads for each metagenome refers to the number of reads after quality and fish host filtering for the fish samples, and after quality filtering for the seawater samples.

The results showed little overlap between the gill and seawater microbiomes, with just one clade out of 94 detected in both environments (Fig 2). The dichotomy between the two environments was particularly striking for one Burkholderiaceae lineage (Burkholderiaceae-B in the release R214 of the Genome Taxonomy Database (GTDB) that we used). This lineage was detected in 118 out of 233 gill samples across a diversity of hamlet species and locations, and all sampling years (2004–2017) but in none of the 19 seawater samples, indicating high prevalence and strong enrichment in the gills compared to seawater.

The reef water samples were collected on the same year and reefs as 22 of our gill samples but not at the same time, so part of the difference between the reef water and gill microbiomes may arguably be due to temporal variation. Yet the reef water samples were collected at several time points over a three-months period, and thereby integrate some temporal variation. Furthermore, Fig 2 indicates that the microbial composition of the reef water is distinct not just from the gill samples that were collected on the same reefs and year but from the gill samples from all fish host species, location and years, indicating a difference that is stable over time, space and fish host species. Similar results were obtained with another gene (*rpL13*, S7 Fig), indicating that this pattern is not marker-specific.

These results stand in stark contrast to previous studies that showed more overlap between gill and seawater microbiomes [8,9,42]. This may be due in part to the fact that the metagenomic approach based on the ribosomal protein loci that we used provides greater variability and resolution than metabarcoding based on the 16S rRNA gene [43], which may have contributed to resolve taxa that were lumped with metabarcoding. Furthermore, the hamlets are not filter-feeders, a feeding mode that is expected to result in higher occurrence of seawater material in the gills and therefore a higher similarity between the two microbiomes [44]. In this regard gill tissue samples were rinsed with sterile water prior to DNA extraction to remove salt from the DMSO buffer in which they were preserved. On one hand, this may have contributed to reducing contamination from seawater. On the other hand, this may have contributed to flushing microbes that are associated with the external gill epithelium, and the drop in osmotic pressure may have burst open microbial cells at the surface of the tissue. Thus, while we expect our gill metagenomes to be highly enriched in gill-associated microbes, they may be biased towards putative endosymbionts and against loose external epithelium associations. Finally the gill and seawater samples originate from tissues versus filters, respectively, and were preserved and extracted following different protocols that are optimized for these two types of samples, which might have contributed to inflate the difference between the two sets of samples.

The EcoPhylo analysis also showed a lower diversity of the gill microbiome compared to coral reef water, which is consistent with previous studies [8,45] (but see [9]), suggesting a specialized microbiome in the gills. Functional analysis of the gill versus reef water metagenomes showed that the gill metagenomes are enriched in categories related to the mobilome, defense mechanisms and cell motility, while the reef water metagenomes are enriched in categories associated to energy production, nucleotide metabolism, carbohydrate metabolism and replication/repair (S2 Table and S8 Fig). This broadly reflects the metabolic requirements of gill-associated versus free-living microbial populations, (e.g., cell motility is essential to colonize the host [46]). All in all, our analyses point to a specific gill microbiome that is less diverse and distinct from seawater, underscoring the particular selective pressures that characterize this specific habitat. This raises the question as to how the microbiome colonizes the gills. Horizontal transmission is likely since fertilization is external and both eggs and larvae are pelagic in the hamlets. This would imply that the taxa identified in the gills are present in the environment, albeit below detection level in the seawater samples that we analyzed.

### A diversity of MAGs were assembled, most of which belong to novel species

A total of 70 MAGs (Metagenome-Assembled Genomes) were assembled from the dataset (Table 1). The average assembly completeness across all retained MAGs was 81.6% (min. 42.2%, max. 99.3%), and the average contamination was 1.4% (min. 0%, max. 7.6%). The MAGs belonged to three bacterial phyla (Pseudomonadota, Chlamydiota and Bacteroidota), four classes (Gammaproteobacteria, Chlamydiia, Alphaproteobacteria and Bacteroidia), seven orders and ten families according to the GTDB, and the phylogenomic analyses below indicate that they represent 17 distinct bacterial lineages. Following a 95% Average Nucleotide Identity (ANI) cut-off [47–49], 15 of these 17 lineages would represent new species. Some of these may also represent new genera, and nine MAGs in the class Alphaproteobacteria could not be assigned to any known order. The fact that the vast majority of gill-associated MAGs belong to novel lineages reinforces our finding that the gill microbiome is distinct from seawater, as seawater is a medium that has been extensively sampled for metagenomic analysis. Furthermore, the EcoPhylo analysis indicates that despite the extensive sampling and sequencing effort, the bacterial lineages that were recovered as MAGs from the gills represent just 54% of the lineages detected by the *rpL16* marker in the gills (Fig 2).

**Table 1:** The 70 Metagenome-Assembled Genomes (MAGs) recovered in this study. ANI: Average Nucleotide Identity with the most closely related species. The host species and location are abbreviated in the MAG names as described in S5 Fig caption.

					CheckM output		Anvio output				
Order	Family Genus	ANI	MAGs	ENA Accession	Comple- teness	Conta- mination	Total Length	Num Contigs	Num Genes (prodigal)	L50	N50
Burkholderiales	f Burkholderiaceae	71.5%	MAG01_puepri_2017	ERS30606204	78.55	2.17	1736968	298	1845	72	7806
		71.3%	MAG02_unipri_2017	ERS30606302	77.58	3.41	1779736	471	1995	118	4674
		71.5%	MAG03_gutpri_2017	ERS30606303	82.37	2.67	1756048	293	1823	78	7400
		71.3%	MAG04_uniflk_2017	ERS30606306	65.54	2.23	1421766	338	1567	85	5351
		71.2%	MAG05_gemflk_2017	ERS30606466	43.51	0.06	842062	185	917	49	5838
		71.3%	MAG06_abehon_2006	ERS30606467	73.87	3.29	1628678	380	1769	96	5529
		71.5%	MAG07_puehon_2006	ERS30606468	86.51	4.12	1925551	435	2105	100	5741
		71.7%	MAG08_maybel_2017	ERS30606469	86.59	2.7	1789012	337	1892	70	7629
		71.3%	MAG09_unibel_2005	ERS30606470	79.99	2.38	1639220	314	1747	75	7139
		71.4%	MAG10_nigbel_2017	ERS30606471	81.50	1.95	1698088	308	1793	71	7465
		71.6%	MAG11_nigboc_2017	ERS30606472	72.98	3.24	1607688	220	1659	51	9978
		71.2%	MAG12_uniboc_2017	ERS30606474	85.44	2.99	1839743	385	1965	86	6499
		71.5%	MAG13_uniboc_2004	ERS30606475	63.72	1.45	1286563	242	1338	63	6757
		71.2%	MAG14_ranhon_2006	ERS30646523	80.42	7.6	1910453	496	2150	127	5009
		71.8%	MAG15_puebel_2017	ERS30646524	46.17	1.40	1128627	263	1294	61	5707
		71.2%	MAG16_unihon_2006	ERS30646525	76.66	3.19	1569097	295	1687	75	7224
		71.2%	MAG17_pueboc_2005	ERS30646526	88.40	2.13	1853929	241	1864	57	10161
		72.1%	MAG18_puebel_2004	ERS30646527	62.92	2.21	1308044	255	1393	61	7110
		71.4%	MAG19_unibel5_2004	ERS30646528	77.02	1.63	1510003	281	1607	72	7212
		71.7%	MAG20_puebel49_2010	ERS30646529	72.25	5.6	1608695	473	1822	126	4185
		71.1%	MAG21_pueboc_2004	ERS30646530	79.20	4.32	1671858	307	1778	70	7405
		71.3%	MAG22_gumhon_2006	ERS30646531	74.49	2.26	1646750	334	1751	81	6609
		71.4%	MAG25_abeboc_2017	ERS30646535	80.48	3.44	1675426	289	1754	73	7370
		71.1%	MAG60_gemare_2017	ERS30646532	84.50	1.50	1766283	279	1840	71	7980
				71.0%	MAG61_abeare_2017	ERS30646534	91.21	1.17	2007638	146	1958	35	18774
		70.9%	MAG62_pueare_2017	ERS30646533	89.91	2.15	1948637	239	1988	53	11882
		70.9%	MAG63_abebel_2017	ERS30646536	89.92	0.35	2061243	118	2020	25	27812
		71.7%	MAG64_sp1pri_2017	ERS30646537	60.60	2.64	1383190	238	1436	61	7357
	f 2013Ark19i	<70%	MAG23_chlpri_2017	ERS30646539	95.12	0	1786673	60	1762	10	51355
	g Ichthyocystis		MAG24_puepri_2017	ERS30646540	82.32	0	1433162	32	1412	6	69897
Pseudomonadales	f Endozoicomonadaceae g Endozoicomonas	91%	MAG26_puepri_2017	ERS30646541	78.08	3.71	3382807	544	3471	144	7393
		92%	MAG27_puebel_2010	ERS30646542	52.67	0.22	2612428	275	2587	59	12820
		> 95%	MAG29_ranhon_2006	ERS30646559	42.19	0.27	2463467	207	2368	48	17573
	f HTCC2089	84-85%	MAG30_chlpri_2017	ERS30646561	85.19	0.93	2070300	93	2068	19	34071
			MAG31_unipri_2017	ERS30646560	48.34	1.67	1072664	601	1472	200	1826
	g UBA4421		MAG65_gemare_2017	ERS30646562	89.85	2.82	2423492	340	2538	86	9449
Chlamydiales	f Simkaniaceae	74.5%	MAG32_puebel49_2010	ERS30646579	88.71	0.25	1836497	106	1771	23	28244
		87.6%	MAG28_gutpri_2017	ERS30646580	95.49	0.56	1889289	145	1868	28	21687
		74.6%	MAG33_puebel49_2010	ERS30646581	54.32	0	1144471	49	1105	11	37229
		74.6%	MAG34_puepri_2017	ERS30646582	94.26	0.23	2186903	48	2054	8	80716
		74.6%	MAG35_abepri_2017	ERS30646583	96.28	0.23	2159716	40	2052	7	85686
		74.6%	MAG36_chlpri_2017	ERS30646584	96.96	0.56	2271777	43	2143	8	86085
		74.6%	MAG37_sp1pri_2017	ERS30646585	96.28	0.56	2374337	47	2225	9	80716
		74.6%	MAG38_unipri_2017	ERS30646586	96.28	0.56	2345327	43	2197	7	117598
		95%	MAG39_nigboc_2017	ERS30646587	96.28	0.23	1903837	52	1792	13	51525
		87.5%	MAG40_chlpri_2017	ERS30646588	96.28	0.23	1957925	15	1832	4	229850
		87.5%	MAG41_puepri_2017	ERS30646589	96.28	0.23	1977536	16	1868	4	234347
		87.5%	MAG42_unipri_2017	ERS30646590	96.28	0.23	1966231	24	1891	4	168832
		84%	MAG43_puebel49_2010	ERS30646591	91.55	2.62	2349060	175	2395	24	28523
Rhizobiales	f WTKA01	69%	MAG44_unipri_2017	ERS30646592	82.49	1.40	1930967	211	1949	53	12289
		69%	MAG45_puepri_2017	ERS30646593	94.65	2.94	2533695	213	2560	39	20418
		69%	MAG67_abeboc_2017	ERS30646594	96.11	0.80	2391179	116	2303	23	31564
G023898725	f G023898725	71.6%	MAG46_puebel49_2010	ERS30646595	88.17	0	1103668	36	1093	11	39442
Alphaproteobacteria			MAG47_puebel49_2010	ERS30646596	93.41	1.10	1036302	65	976	9	35745
			MAG48_pueboc12	ERS30646597	76.96	0.20	844125	332	1010	91	3021
		68%	MAG49_puepri_2017	ERS30646598	91.21	1.10	1042572	27	973	7	59438
			MAG50_sp1pri_2017	ERS30646599	90.11	1.10	1006234	26	932	5	72090
			MAG51_unipri_2017	ERS30646600	91.21	1.10	980855	27	895	7	54460
			MAG66_gutpri_2017	ERS30646601	56.03	0.00	508441	132	552	44	4168
			MAG68_nigbel_2004	ERS30646602	82.34	0.26	832208	225	921	59	4684
			MAG69_niggun_2005	ERS30646603	93.41	1.10	994405	54	922	11	32279
			MAG70_puegun_2005	ERS30646604	86.81	0.00	868873	126	867	29	9773
Rickettsiales	f Midichloriaceae	93.4%	MAG52_abehon_2006	ERS30646605	62.05	0.55	1139219	588	1524	185	1981
		94.15%	MAG53_nigboc_2017	ERS30646606	97.8	0	1279092	83	1291	20	21258
	f Rickettsiaceae	68%	MAG54_puebel49_2010	ERS30646607	95.02	0.47	1027183	34	849	7	46192
	f Flavobacteriaceae g	≈78%	MAG55_unipri_2017	ERS30646611	91.23	0.71	1931275	104	1927	20	25545
Flavobacteriales	Arcticimaribacter		MAG56_chlpri_2017	ERS30646612	94.91	0.47	2238297	99	2239	19	36830
	f Flavobacteriaceae	< 72%	MAG57_puebel49_2010	ERS30646613	94.12	0	2040907	207	2047	49	13745
	g MS024-2A		MAG58_unipri_2017	ERS30646614	99.26	0	2273742	160	2259	35	22769
			MAG59_abepri_2017	ERS30646615	98.9	0	2204823	162	2173	36	22095

We focused on the Burkholderiales, Pseudomonadales, Rhizobiales and Flavobacteriales for more in-depth analysis, which represent 63% of the MAGs that were recovered. The results of the phylogenomic analyses for these orders are presented in S10-S20 Figs. Some MAGs were related to fish gill pathogens, notably *Ichthyocystis sparus* and *I. hellenicum* [23] in the Burkholderiales (S10 Fig), as well as *Endozoicomonas sp900299555* and *E. elysicola* [22,50] in the Pseudomonadales (S12 Fig). The genus *Endozoicomonas* is known for its association with corals, but also includes pathogenic and parasitic lineages of other marine animals [51]. Other MAGs from this genus were identified as close relatives of *E. atrinae*, which was isolated from the gut of the comb pen shell (*Atrina pectinata*) [52]. Other MAGs related to predominantly free-living seawater genera, notably *UBA4421* in the Pseudomonadales (S14 Fig) as well as *MS024-2A* and *Arcticimaribacter* in the Flavobacteriales (S18 Fig). Finally, some MAGs were found to be related to *WTKA01 sp011524605* in the Rhizobiales, which is associated with coral reef macroalgae biofilm (S16 Fig). All in all, these results indicate that the microbes found in the gills are related to bacteria that are phylogenetically diverse and have a variety of lifestyles (symbionts, pathogens, free-living and biofilm-associated), indicating that the gill microbiome was assembled from a collection of distinct eco-evolutionary trajectories.

### A novel lineage in the Burkholderiaceae family is particularly prevalent

The Burkholderiaceae lineage that was most prevalent in the gills according to the EcoPhylo analysis (Fig 2) represented 28 of the 70 MAGs across ten fish host species, six sampling locations and five sampling years between 2004 and 2017 (Table 1). This was followed by one Alphaproteobacteria species represented by 9 MAGs, confirming the high prevalence of the Burkholderiaceae lineage. Considering that this lineage is novel following a 95% ANI cut-off, its high prevalence in the gills but absence in seawater according to the EcoPhylo analysis (Fig 2), and the fact that the gill dataset encompasses different sampling expeditions, DNA extraction batches and sequencing runs over several years, it is unlikely that it represents a contamination. Furthermore, we analyzed 67 fin clips from different fish hosts and locations that were extracted and sequenced following the same procedure as the gills as controls, and recovered just six MAGs with >40% completeness from these samples. These included three *rpL16* clades (one *Vibrio* that was also found in the gills and three Alphaproteobacteria) but not the Burkholderiaceae lineage (Fig 2), ruling out a systematic contamination of the hamlet samples by the Burkholderiaceae lineage. Finally, we did not recover the Burkholderiaceae lineage in gill samples from *Serranus tortugarum* and *Serranus tabacarius*, the hamlets closest relatives, as well as *Serranus tigrinus*, which points to a specific association.

**Fig 3:**
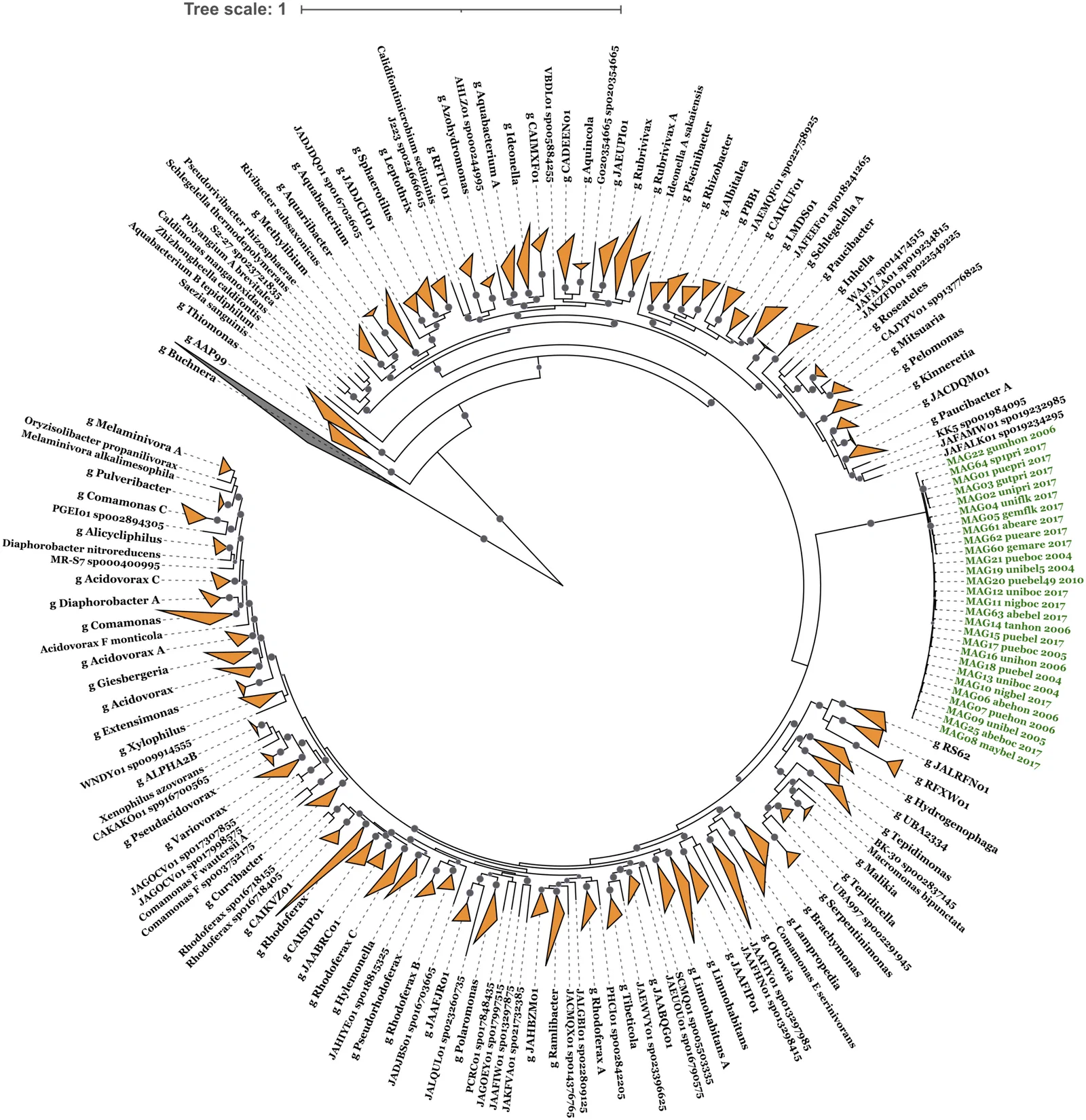
Maximum-likelihood phylogenomic placement of the MAGs from the most prevalent lineage in the Burkholderiaceae family. These 28 MAGs (in green) form a distinct clade that is ~70% diverged from the most closely related species based on average nucleotide identity. Genus names are indicated by a leading “g” where genus nodes were collapsed. Triangles size represent the number of genomes in these collapsed nodes. The tree is rooted with the genus *Buchnera* (in black). Bootstrap values represented by gray circles, only bootstrap >95 are shown.

Phylogenomic analysis indicated that the Burkholderiaceae MAGs form a deeply branching clade in the family relative to known species (Fig 3). The ANI values to the most closely related species ranged between 70.9% and 72.1% (Table 1), and the identity of the most closely related species varied from MAG to MAG. Furthermore, these ANI comparisons were based on the genomic regions that did align, which represented a small proportion of the genome (≤16.5%), indicating that the Burkholderiaceae MAGs are distantly related to any known species in the family. The 28 Burkholderiaceae MAGs shared 95.7–98.5% ANI and had a mean alignment fraction of 74.2%, indicating that they would belong to the same species following a 95% ANI and a 50% alignment fraction threshold [47–49,53].

**Fig 4:**
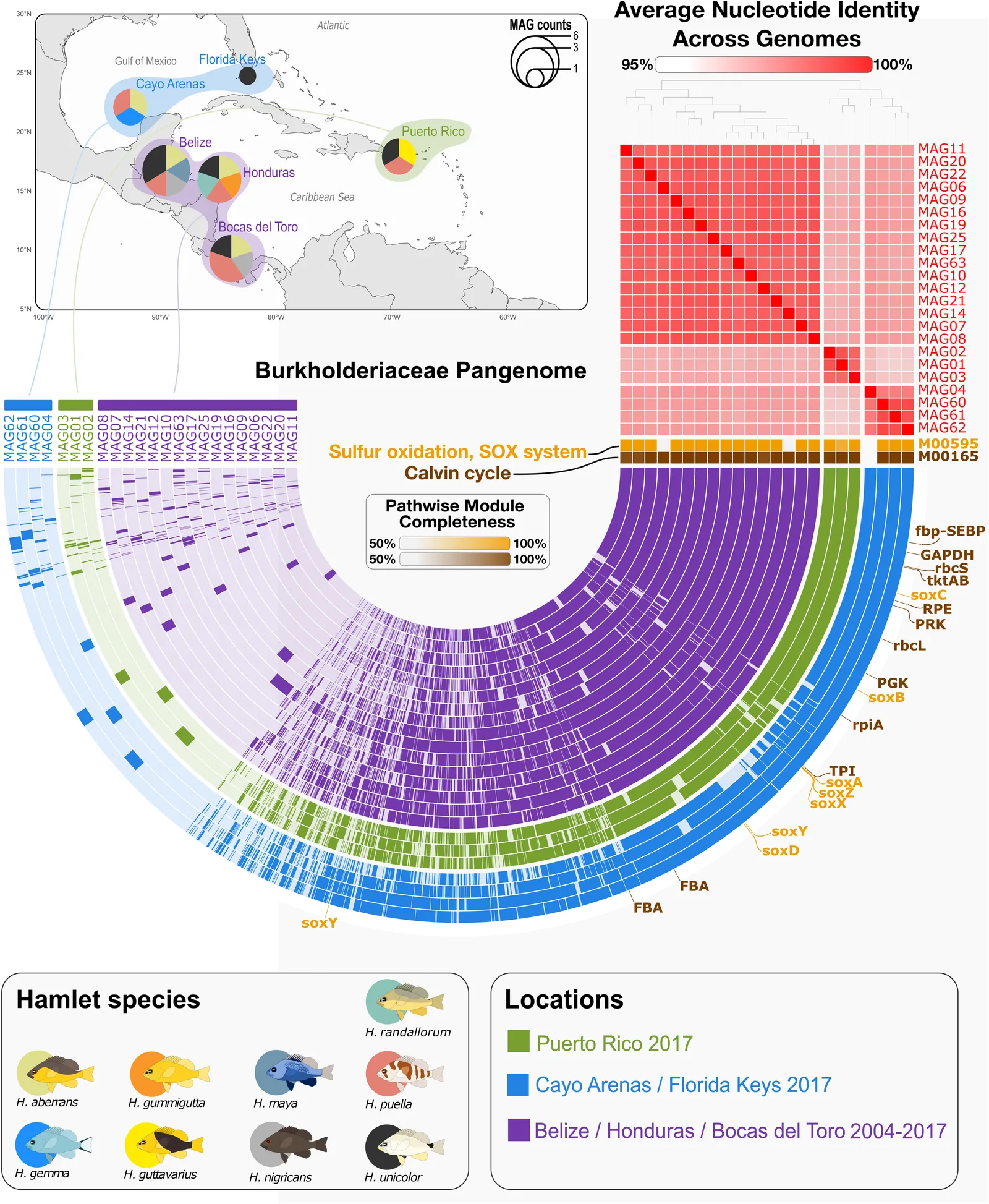
Pangenome of the most prevalent Burkholderiaceae lineage. Each layer represents one MAG. The heatmap in the top-right shows full average nucleotide identity among all genomes. A total of 2797 gene clusters were identified, including 490 core gene clusters (recovered in all genomes), 1732 accessory gene clusters (recovered in only part of the genomes) and 575 singleton gene clusters (recovered in just one genome). A heatmap of pathwise module completeness scores for the Sulfur oxidation module (yellow) and the Calvin cycle module (brown) is shown below the ANI heatmap. Gene clusters containing the genes belonging to these modules are indicated by gene name labels around the outside of the pangenome. For the Calvin cycle, these genes include fbp-SEBP: bifunctional fructose-1,6/sedoheptulose-1,7-bisphosphatase; rbcL: ribulose-bisphosphate carboxylase, large chain; rbcS: ribulose-bisphosphate carboxylase; small chain; tktAB: transketolase; RPE: ribulose-phosphate 3-epimerase; GAPDH: glyceraldehyde-3-phosphate dehydrogenase; PRK: phosphoribulokinase; PGK: phosphoglycerate kinase; rpiA: ribose-5-phosphate isomerase; FBA: fructose-bisphosphate aldolase; and TPI: triosephosphate isomerase. Note that enzymes TPI and RPE are not included in the version of KEGG module used in our analysis and were manually identified. For the SOX system, these genes include the sulfur-oxidizing proteins soxABXYZ and the S-disulfanyl-L-cysteine oxidoreductases soxCD. Basemap from the R package rnaturalearth (Natural Earth Admin 0 – Countries dataset, large scale, https://www.naturalearthdata.com).

The high number of Burkholderiaceae MAGs allowed us to generate a pangenome for the group (Fig 4). It revealed broad-scale geographic structure, with samples from the Gulf of Mexico, eastern Caribbean and western Caribbean grouping together regardless of fish host species and collection year. This pattern was confirmed by phylogenomic analysis (S9 Fig). It reflects the phylogenomic structure observed in the fish host radiation, which is poorly structured with regard to fish host species but shows a biogeographic signal among the same three regions [33]. These results parallel what has been observed in lucinid clams, where some chemosynthetic symbionts show low host specificity but geographic partitioning [54].

### The most prevalent Burkholderiaceae lineage has chemosynthetic potential

Functional analysis of the 28 Burkholderiaceae MAGs identified the complete set of Calvin cycle enzymes for carbon fixation (Figs 4 and S21 and S3 Table), indicating autotrophic potential. This is in line with the observation that many members of the Burkholderiaceae host a complete Calvin cycle [55]. Genes involved in the dark phase of the Crassulacean Acid Metabolism (CAM) pathway were also present, but in bacteria these genes might contribute to other functions than carbon fixation as they do in plants. Genes encoding the full Sox complex (soxABCDXYZ) for aerobic thiosulfate oxidation were also recovered. The Calvin cycle and thiosulfate oxidation pathways were recovered complete not only in MAGs co-assembled from multiple fish hosts, but also in MAGs assembled from a single fish host, indicating that they co-occur not only in the same MAG but also in the same fish host. Taken together, the Calvin cycle and thiosulfate oxidation pathways provide potential for chemosynthesis, chemolithoautotrophy more specifically. Chemolithoautotrophic thiosulfate oxidizers have been reported in the Burkholderiales, albeit in different ecological contexts. They have notably been identified in the soil rhizosphere [56], and these were facultative chemolithoautotrophs. In our case the lack of a complete glycolysis pathway would suggest that chemolithoautotrophy is obligate, but on the other hand the occurrence of a complete TCA cycle contrasts with most other obligate chemoautotrophs.

Experimental evidence indicates that the balance between the production and oxidation of hydrogen sulfide in the gills may serve as an oxygen sensing mechanism for the fish host [57]. The mitochondrial catabolism of hydrogen sulfide by the fish host involves a series of oxidation steps, with thiosulfate as an intermediate product [58]. On the basis of these observations, we speculate that thiosulfate oxidation might be linked to oxygen sensing in the gills. It is nevertheless not clear how exactly the fish host and the gill microbiome would interact in this context.

Sulfide:quinone oxidoreductase, which is essential to link reduced sulfur compounds to the quinone pool, was recovered in 21 of the 22 MAGs (S4 Table). Furthermore, respiratory electron transport chain modules comprising NADH:quinone oxidoreductase (Complex I), cytochrome bc1 complex, and cytochrome c oxidase were recovered complete in 14 of 22 MAGs, while the remaining MAGs were incomplete for at least one component (S5 Table). This suggests that reduced quinones may transfer electrons to cytochrome c, which are then passed to oxygen via a terminal cytochrome c oxidase, indicating capacity for aerobic respiration. Additional bd-type quinol oxidases were also recovered in 15 of the 22 MAGs based on the presence of both core subunits (cydAB), with 13 MAGs also encoding the accessory subunit cydX, suggesting ability to cope with microaerobic conditions (S4 Table). Enzymes related to taurine breakdown were identified in all 22 MAGs, with a complete taurine transport system present in 20 of 22 MAGs, while glycine betaine/proline uptake systems were present in 22 of 22 MAGs (S4 Table). Beyond their functions as osmolytes, taurine may contribute to sulfur cycling in the gills [59] and glycine betaine has been suggested as another possible source of carbon in symbiotic interactions [60].

The high prevalence of the Burkholderiaceae lineage suggests that it is a predominant constituent of the hamlet core gill microbiome. In this regard it may also be important for niche occupation, contributing to stabilize the gill microbiome and limit colonization by pathogens. This hypothesis is consistent with the occurrence of components of the CRISPR-Cas immune system in its genome, which is essential for defense against phage invasions [61] and has been suggested to enhance the resilience of sponge microbiomes [62]. More generally, the Burkholderiaceae lineage exhibited diverse metabolic capacities, encoding 56 metabolic modules recovered at ≥75% completeness in at least one MAG, including 10 amino acid biosynthesis modules (S21 Fig) as well as a variety of ATP-binding cassette (ABC) transporters, cofactors and vitamins. Fifty-six metabolic modules is within the range found in the other MAGs that we analyzed (36–45, S23–S28 Figs), but higher than the range found in the Chlamydiales, Rickettsiales, G023898725 and Alphaproteobacteria MAGs that are not the focus of this study (13–24). Furthermore, the functional analyses in the Burkholderiaceae were only conducted on MAGs with completeness ≥70% (S21 Fig), yet even within this subset there was still a positive correlation between MAG completeness and number of metabolic modules (S22 Fig), indicating that we may have missed metabolic modules notwithstanding the large sequencing effort.

Associations between sulfur-metabolizing bacteria and gills have been reported before, but in invertebrates and often in extreme environments such as deep-sea hydrothermal vents or sediments that are rich in reduced sulfur compounds [63–65]. In our case the functional significance of the chemosynthetic potential of the Burkholderiaceae lineage for the fish host or other members of the gill microbiome remains to be established. While the hamlets live in shallow-water coral environments that are not particularly extreme in terms of environmental conditions, the gills may nonetheless provide a specific microhabitat that is enriched in CO_2_ and other compounds generated by the respiration and excretion of the host, which may be conducive to chemosynthesis. All in all, our metagenomic data indicate metabolic potential for chemosynthesis in the most prevalent species in the hamlet gills. Considering that the hamlets are not particularly specialized ecologically, we speculate that such associations may be widespread in fishes.

### The gill microbiome harbors a variety of metabolic functions

The results of the functional analyses for the Burkholderiales, Pseudomonadales, Rhizobiales and Flavobacteriales are presented in S21 and S23-S28 Figs. Metabolic modules for carbon fixation were also identified in the Rhizobiales, which here again is in line with the observation that many members of this order host a complete Calvin cycle [55]. We also recovered a number of modules involved in nitrogen cycling. These include nitrate assimilation and dissimilatory nitrate reduction into ammonia (both in *Endozoicomonas*). The nitrogenous waste of the fish host is excreted as ammonia in the gills, and experimental evidence indicates that the gill microbiome can contribute to ammonia detoxification through ammonia oxidation and denitrification [11]. We did not identify modules that relate to these specific functions in the MAGs that we functionally analyzed, but ammonium transporters were nonetheless detected in almost half of the MAGs, suggesting active ammonia transport into cells [66]. The nitrogen regulatory protein GlnK, a member of the PII family, was found in 25 of 28 MAGs from the Burkholderiaceae lineage and in 13 additional MAGs. GlnK regulates ammonium assimilation by interacting with the ammonium transporter AmtB, a process modulated by uridylylation via the GlnD enzyme, which was found in 18 of 28 MAGs from the Burkholderiaceae lineage and in eight additional MAGs [66,67].

We also identified several COG24 functional categories associated with colonization and interactions between the fish host and microbes across pathogenic, commensal and mutualistic relationships. These include eukaryotic-like proteins such as ankyrin repeats and tetratricopeptide repeats, as well as membrane proteins involved in the export of O-antigen and teichoic acid. Several MAGs also encoded genes for type IV pili that provide potential for biofilm formation and host cell adherence [68], as well as flagella construction and chemotaxis signaling (Burkholderiaceae MAGs lineage not included), indicating motility potential (S6 Table).

Our results further suggest a complex web of potential nutritional interactions within the gill microbiome, notably through the biosynthesis of a diverse array of B vitamins. These include riboflavin (B2), folic acid (B9), pantothenic acid (B5), pyridoxamine (B6), niacin (B3) and cobalamin (B12) across various MAGs. The ability of different MAGs to produce essential vitamins suggests possible beneficial relationships both between microbes and between the microbiome and the host, as suggested for Pseudomonadales in octocorals [69] and gammaproteobacterial symbionts in deep-sea bivalves [70]. Beyond vitamins, the gill MAGs harbored modules for the production of diverse metabolites. For example ectoine (in *Endozoicomonas*), an osmolyte that has been suggested to contribute osmoregulation in marine fishes [71].

## Conclusion

To the best of our knowledge, the metagenomic results presented here constitute the first line of evidence suggesting that fishes may host sulfur-oxidizing chemosynthetic bacteria in their gills. Fluorescent In-Situ Hybridization (FISH) is needed to determine the location and abundance of the Burkholderiaceae bacteria in the hamlet gill tissue. Gene expression analysis would provide a basis to asses the extent to which chemosynthetic potential is realized *in vivo*. If so, experimental work will be needed to assess whether it relates to the physiology of the fish host or other gill microbes. While our metagenomic analysis constitutes an advance in the characterization of the fish gill microbiome, what it really highlights is how little is known about its diversity and potential fish host-microbe and microbe-microbe interactions, which include not only bacteria but also viruses, archaea, fungi and protists. The high proportion of novel taxa identified indicates that we are still at an exploratory and discovery phase. In this regard more sequencing across a broader diversity of fishes and habitats is required. More generally it is time for concerted research aimed at disentangling the complex web of interactions that take place in the fish gill microbiome. The metagenomes described here lay a foundation for this work by allowing to develop species-specific markers that can be used for targeted research and establish the metabolic capabilities of specific microbes to culture them.

## Materials and methods

### Sample collection and processing

This study includes a total of 353 shotgun-sequenced gill tissue samples from 15 hamlet species collected between 2004 and 2017 at eight locations across the Greater Caribbean (Figs 1 and S1 and S7 Table). This represents a total of 60 fish host species/location/collection year combinations, with a median sample size of 3.5 samples per fish host species/location/collection year (min. one, max. 49). Furthermore, we considered 67 fin clip samples from different hamlet species and locations that were extracted and sequenced together with the gill samples and following the same procedure. The objective was not to characterize the skin microbiome—swabs would have been more appropriate for that—but to process and analyze another type of hamlet tissue sample in the same way as the gills, serving as controls. We also analyzed three gill samples from *Serranus tortugarum* and two from *Serranus tabacarius*, the hamlets closest relatives, as well as two gill samples from *Serranus tigrinus*. All these genomes have been previously published in studies on the fish host [32–36]. Finally, we considered 19 shotgun-sequenced reef water samples that were collected in Bocas del Toro (Panama) in 2017 [39,40]. These samples provide the opportunity to compare reef water microbiomes with gill microbiomes, in particular the 61 gill samples that were collected on the same reefs in Bocas del Toro. Twenty-two of these gill samples were also collected in 2017, albeit not at the exact same time as the reef water samples.

Briefly, fishes were collected on scuba using either microspears or hook-and-line. Gill or fin tissue samples were dissected, preserved in salt-saturated DMSO and stored at 4 °C. Bulk DNA was extracted from about 2 mm^2^ of tissue using Qiagen MagAttract High Molecular Weight kits, without any particular step for microbial DNA enrichment or fish host DNA depletion. Extracted DNA was shotgun-sequenced on an Illumina platform (2 × 151 bp) at a coverage of approximately 20x with respect to the fish host genome. Fish host reads were identified by mapping them to the fish host reference genome with bowtie2 [72] and filtered (note that the fish host genome assembly procedure included a microbial DNA decontamination step [34]). Reef water samples were filtered through 0.22 µm nitrocellulose membranes and stored at -80°C. DNA was extracted with a Qiagen Powersoil extraction kit and sequenced on an Illumina platform [39,40].

### Taxonomic composition of the gill microbiome

We started by characterizing the taxonomic composition of the gill microbiome using the read classification tool Kraken2 v2.17.1 [73] with the PlusPF Kraken2 database. The output from Kraken2 was further processed with Bracken v3.0.1 [74], which uses a Bayesian approach to re-estimate abundances after classification by Kraken. Reads classified as animals and major photosynthetic eukaryotic lineages were excluded, along with reads assigned to PhiX, which was used as a sequencing control. The taxonomic composition of the gill microbiome was analyzed at the phylum, class, order and family levels and displayed with ggplot2 [75] v3.4.3 in R. Differences in gill microbiome composition were tested using marginal PERMANOVA (adonis2, vegan package v2.6.4; 999 permutations) [76] based on Bray–Curtis dissimilarities calculated from relative abundance data aggregated at the phylum level. The analysis assessed the effects of sequencing batch, biogeographic region, geographic location, fish host species and sampling year, and additional models including interaction terms were used to test species–location and species–year interactions. Patterns of community similarity were visualized using principal coordinates analysis (PCoA).

### Metagenome assembly

The entire metagenome assembly workflow, from sequence quality control to binning, was conducted on the anvi’o 7.1 platform following the snakemake workflow (https://merenlab.org/2018/07/09/anvio-snakemake-workflows/). The assessment of raw sequence quality was performed with the iu-filter-quality-minoche script [77]. Subsequently, sequences were assembled into contigs with Megahit v1.2.9 [78], with a minimum contig length of 1000 bp. For the 46 fish host species/locations/collection year for which we had more than one sample, metagenomes were co-assembled per fish host species, location and collection year. Gene prediction was conducted with Prodigal v2.6.3 [79] and single-copy genes were identified with HMMER v3.2.1 [80]. Quality-filtered reads were mapped to the assembled contigs with Bowtie v2.3.5 [72] and SAMtools v1.10 [81]. The contigs were then binned into Metagenome-Assembled Genomes (MAGs) with anvi-cluster-contigs using the unsupervised binning tool CONCOCT v1.1.0 [82]. A subsequent supervised binning procedure was executed through the anvi-interactive interface of anvi’o v7.1. Specifically, the MAGs were inspected and refined using anvi-refine. For the 14 fish host species/location/collection year for which we had only one sample (S1 Fig), MAGs were binned from single assemblies with MetaBAT2 v2.12.1 [83] after assembling contigs with Megahit v1.2.9 [78]. Only bins with a completion rate of at least 40% and contamination levels below 10%, as assessed by CheckM v1.2.2 [84], were retained for further analysis. The same assembly procedure was used for the gill, fin clip and seawater samples.

### Gills versus seawater

The EcoPhylo workflow implemented in anvi’o v9-dev (https://anvio.org/help/main/workflows/ecophylo/) was used to profile the distribution of microbial populations across metagenomes from gills, fin clips and published metagenomes from seawater collected over coral reefs in Bocas del Toro (Panama) in 2017 [39,40]. This is one of the locations where the gills considered in this study were collected (in 2004, 2005 and 2017). Briefly, this workflow [85] (1) identifies gene family homologs in MAGs and metagenome assemblies using a profile Hidden Markov Model (HMM) [80], (2) clusters open reading frames and selects representative non-redundant sequences with mmseqs2 v13.45111 [86], (3) uses the representative nucleotide sequences for metagenomic read recruitment to profile the ecological distribution of the protein family, and (4) calculates a phylogenetic tree with the translated open reading frames to explore the phylogenetic diversity of the protein family [72,87–90]. Lastly, the workflow displays an interactive figure to explore the phylogeography of the protein family.

We used the ribosomal protein single-copy core gene *rpL16* as a proxy for the presence of a microbial population. This gene was recovered in 62 of the 70 MAGs, and all the MAG lineages were represented by at least one MAG containing the gene. Gene sequences from MAGs and metagenomic assemblies were used for read recruitment to assess detection, the proportion of a contig that is covered by at least one read (https://merenlab.org/2017/05/08/anvio-views/). A detection threshold of 70% was used to establish the presence of a ribosomal protein in a metagenome and we adjusted the EcoPhylo workflow with the following parameters: cluster-X-percent-sim-mmseqs -min-seq-id parameter set to 95% and -cov-mode to 1. Finally, we repeated the analysis with *rpL13* (recovered in 59 of the 70 MAGs) to assess the consistency of the results with another phylogenetic marker.

### Phylogenomic analysis

Following interactive refinement in anvi’o v9-dev, MAGs were exported with anvi-summarize for taxonomic classification. Taxonomic assignments were conducted with the Genome Taxonomy Database Toolkit (GTDB-Tk) v2.3.2 using reference data vr214 [91,92]. The classify workflow was applied using a collection of 120 bacterial gene markers. This involved placing the MAGs in a reference tree based on the Genome Taxonomy Database (GTDB) with FastANI v1.33 [49] and pplacer v1.1.alpha19 [93] to obtain a first approximate phylogenetic placement of the MAGs. Subsequently, the de novo workflow in GTDB-tk v2.3.2 [91] was used to infer the phylogenetic relationships between the targeted MAGs and related reference genomes. In this workflow, the infer step used FastTree v2.1.11 [89] with the WAG+GAMMA model to build *de novo* trees. The trees are then rooted with a specified outgroup and labeled with the GTDB taxonomy. The anvi-compute-genome-similarity tool v9-dev (https://anvio.org/m/anvi-compute-genome-similarity) was used with PyANI v.0.2.12 [94] to quantify the genomic similarity between reconstructed MAGs and the most closely related genomes.

### Pangenome

We leveraged the high prevalence of one novel lineage of Proteobacteria in the Burkholderiaceae family (28 MAGs) to generate a pangenome across fish host species, sampling locations and sampling years for this lineage. In order to minimize bias due to MAG contamination and low completeness, we only considered MAGs with a completeness of ≥70% and an average contamination of 2.93%, except for one MAG from the Florida Keys as a representative of this area, resulting in 23 MAGs for this analysis. Functional annotations from multiple sources, including KEGG (Class and Module), KEGG BRITE, KOfam and COG24 (Function, Category and Pathway), were integrated into the genome databases using anvi’o. The script anvi-pan-genome in anvio (v9-dev) was then used with an MCL inflation parameter of 2.0 to identify *de novo* gene families and their distribution across genomes based on amino acid sequence homology determined by DIAMOND v.2.1.8 in BLASTP mode [95]. Subsequently, anvi-display-pan was executed and an ANI heatmap was generated with anvi-compute-genome-similarity. The pangenome visualization was polished with Inkscape (https://inkscape.org/).

### Functional analysis

We functionally annotated the contig databases of all MAGs with KOfam, a customized Hidden Markov Model (HMM) database of Kyoto Encyclopedia of Genes and Genomes (KEGG) orthologs [96], using anvi-run-kegg-kofams (anvio v9-dev) (https://anvio.org/m/anvi-run-kegg-kofams). Note that we used a version of KEGG downloaded in September 2023 (for reproducibility, the hash of the KEGG snapshot available via anvi-setup-kegg-kofams was a2b5bde358bb). We used the KEGG annotations with anvi-estimate-metabolism anvio v9-dev) (https://anvio.org/m/anvi-estimate-metabolism) [97] to reconstruct metabolic modules and estimate module completeness, implementing the -only-complete flag. Module completeness was defined by the proportion of complete steps, with a threshold of at least 0.75 to consider a module complete. Additionally, we used the NCBI Clusters of Orthologous Genes (COGs) with anvi-run-ncbi-cogs (anvio v9-dev) [98] and the European Bioinformatics Institute (EBI) Pfam database [99]. Heatmaps were generated with ggplot2 in R to visualize the completeness of the metabolic modules.

In order to test for functional differences between the gill and reef water microbiomes, we compared functional profiles of the gill and reef water metagenomes from the same location and year (Bocas del Toro 2017) using COG annotations in anvi’o (v9-dev). The COG24 categories assigned per gene were normalized to relative proportions and group differences were tested using two-sample Welch’s t-tests with Benjamini–Hochberg correction for multiple testing.

## Supporting information

S1 Fig Overview of the sampling design. The dataset comprises 360 shotgun-sequenced gill samples representing 18 reef fish species, including 15 hamlets (*Hypoplectrus*) and 3 outgroup serranids (*Serranus*). Hamlets include pue: *Hypoplectrus puella*, uni: *H. unicolor*, nig: *H. nigricans*, abe: *H. aberrans*, aff: *H. affinis*, chl: *H. chlorurus*, flo: *H. floridae*, gem: *H. gemma*, gum: *H. gummigutta*, gut: *H. guttavarius*, ind: *H. indigo*, may: *H. maya*, pro: *H. providencianus*, ran: *H. randallorum*, and sp1: *Hypoplectrus sp. 1*. Outgroup species comprise tor: *Serranus tortugarum*, tab: *Serranus tabacarius* and tig: *Serranus tigrinus*. Samples were collected at eight locations across the Caribbean – Cayo Arenas (are) and the Florida Keys (flk) in the Gulf of Mexico; Belize (bel), Honduras (hon), Bocas del Toro in Panama (boc) and Guna Yala (gun) in the Western Caribbean; and Puerto Rico (pri) and Barbados (bar) in the Eastern Caribbean.
(PDF)

S2 Fig Taxonomic composition of the gill microbiome at the phylum level. Each stacked bar represents one of the 60 host populations (fish host species-location-year combination) and shows the mean relative abundance of bacterial phyla averaged across all samples within that population. Taxonomic assignments are based on genomic read classification. The first three letters of the bar labels correspond to the fish host species (pue: *H. puella*, uni: *H. unicolor*, nig: *H. nigricans*, abe: *H. aberrans*, aff: *H. affinis*, chl: *H. chlorurus*, flo: *H. floridae*, gem: *H. gemma*, gum: *H. gummigutta*, gut: *H. guttavarius*, ind: *H. indigo*, may: *H. maya*, pro: *H. providencianus*, ran: *H. randallorum*, sp1: *Hypoplectrus sp. 1*), and the subsequent three letters refer to Caribbean sampling sites: Cayo Arenas (are), Florida Keys (flk), Belize (bel), Honduras (hon), Bocas del Toro, Panama (boc), Guna Yala (gun), Puerto Rico (pri) and Barbados (bar).
(PDF)

S3 Fig Taxonomic composition of the gill microbiome from read recruitment analysis at the class level. Each stacked bar represents one of the 60 host populations (fish host species-location-year combination) and shows the mean relative abundance of bacterial phyla averaged across all samples within that population. Taxonomic assignments are based on genomic read classification. The first three letters of the bar labels correspond to the fish host species (pue: *H. puella*, uni: *H. unicolor*, nig: *H. nigricans*, abe: *H. aberrans*, aff: *H. affinis*, chl: *H. chlorurus*, flo: *H. floridae*, gem: *H. gemma*, gum: *H. gummigutta*, gut: *H. guttavarius*, ind: *H. indigo*, may: *H. maya*, pro: *H. providencianus*, ran: *H. randallorum*, sp1: *Hypoplectrus sp. 1*), and the subsequent three letters refer to Caribbean sampling sites: Cayo Arenas (are), Florida Keys (flk), Belize (bel), Honduras (hon), Bocas del Toro, Panama (boc), Guna Yala (gun), Puerto Rico (pri) and Barbados (bar).
(PDF)

S4 Fig Taxonomic composition of the gill microbiome at the order level. Each stacked bar represents one of the 60 host populations (fish host species-location-year combination) and shows the mean relative abundance of bacterial phyla averaged across all samples within that population. Taxonomic assignments are based on genomic read classification. The first three letters of the bar labels correspond to the fish host species (pue: *H. puella*, uni: *H. unicolor*, nig: *H. nigricans*, abe: *H. aberrans*, aff: *H. affinis*, chl: *H. chlorurus*, flo: *H. floridae*, gem: *H. gemma*, gum: *H. gummigutta*, gut: *H. guttavarius*, ind: *H. indigo*, may: *H. maya*, pro: *H. providencianus*, ran: *H. randallorum*, sp1: *Hypoplectrus sp. 1*), and the subsequent three letters refer to Caribbean sampling sites: Cayo Arenas (are), Florida Keys (flk), Belize (bel), Honduras (hon), Bocas del Toro, Panama (boc), Guna Yala (gun), Puerto Rico (pri) and Barbados (bar).
(PDF)

S5 Fig Taxonomic composition of the gill microbiome at the family level. Each stacked bar represents one of the 60 host populations (fish host species-location-year combination) and shows the mean relative abundance of bacterial phyla averaged across all samples within that population. Taxonomic assignments are based on genomic read classification. The first three letters of the bar labels correspond to the fish host species (pue: *H. puella*, uni: *H. unicolor*, nig: *H. nigricans*, abe: *H. aberrans*, aff: *H. affinis*, chl: *H. chlorurus*, flo: *H. floridae*, gem: *H. gemma*, gum: *H. gummigutta*, gut: *H. guttavarius*, ind: *H. indigo*, may: *H. maya*, pro: *H. providencianus*, ran: *H. randallorum*, sp1: *Hypoplectrus sp. 1*), and the subsequent three letters refer to Caribbean sampling sites: Cayo Arenas (are), Florida Keys (flk), Belize (bel), Honduras (hon), Bocas del Toro, Panama (boc), Guna Yala (gun), Puerto Rico (pri) and Barbados (bar).
(PDF)

S6 Fig Principal coordinates analysis (PCoA) of gill microbiome community composition at the phylum level. Ordination was based on Bray–Curtis dissimilarities calculated from sample-level phylum relative abundance data derived from genomic read classification. Samples in ordination are colored according to fish host species (top left), sampling year (top right), sampling location (bottom left) and biogeographic region (bottom right). Fish host species are abbreviated as follows: abe: *H. aberrans*, aff: *H. affinis*, chl: *H. chlorurus*, flo: *H. floridae*, gem: *H. gemma*, gum: *H. gummigutta*, gut: *H. guttavarius*, ind: *H. indigo*, may: *H. maya*, nig: *H. nigricans*, pro: *H. providencianus*, pue: *H. puella*, ran: *H. randallorum*, sp1: *Hypoplectrus sp. 1*, and uni: *H. unicolor*. Sampling locations are abbreviated as follows: Cayo Arenas (are), Barbados (bar), Belize (bel), Bocas del Toro, Panama (boc), Florida Keys (flk), Guna Yala (gun), Honduras (hon) and Puerto Rico (pri). Biogeographic regions correspond to the Gulf of Mexico, Western Caribbean and Eastern Caribbean.
(PDF)

S7 Fig Ribosomal protein L13 (*rpL13*) genes assembled from fin clip, fish gill and seawater metagenomes. *RPL13* was used as a proxy for the detection of microbial clades. Rows correspond to metagenomic samples, and columns represent *rpL13* representative sequences clustered at 95% nucleotide identity. Heatmap values indicate presence/absence based on read recruitment (70–100% detection). The heatmap highlights differences between gill and seawater samples, including a highly prevalent clade in gills that is absent from seawater, corresponding to a novel lineage within the Burkholderiaceae family. The green part of the horizontal bar at the bottom indicates whether a given rpL13 represented by a MAG recovered from fish gill metagenomes. Red star denotes gill samples from *Serranus* fish. Gill samples collected Bocas 2017 colored in green and gill samples from Bocas 2004/2005 in purple, both just above seawater samples.
(PDF)

S8 Fig Comparison of COG functional category proportions between Bocas 2017 gill and seawater metagenomes. Violin plots with overlaid boxplots show the distribution of relative abundances of COG functional categories across individual metagenomes from gill and seawater environments, with points representing individual samples. Functional categories are ordered by the difference in mean proportions between environments (gill minus seawater). Asterisks denote statistically significant differences based on two-sample Welch’s t-tests with Benjamini–Hochberg false discovery rate correction (*, padj < 0.05; **, padj < 0.01; ***, padj < 0.001).
(PDF)

S9 Fig Maximum-likelihood phylogenetic tree of 28 MAGs from the most prevalent lineage in the Burkholderiaceae family. The MAGs from Cayo Arenas (are) and the Florida Keys (flk) in the Gulf of Mexico and Puerto Rico (pri) formed a clade diverged from the remaining locations: Belize (bel), Honduras (hon) and Bocas del Toro in Panama (boc).
(PDF)

S10 Fig Maximum-likelihood phylogentic placement of the MAGs in the 2013Ark19i family (order Burkholderiales). The tree is rooted with the genus *Buchnera* (in red). Support values at the nodes represent non-parametric bootstrap values.
(PDF)

S11 Fig Average Nucleotide Identity (ANI) of the MAGs in the 2013Ark19i family (order Burkholderiales). These two MAGs were very closely related (>99% ANI) but showed an ANI < 70% with the other genomes in the family (*Ichthyocystis sparus* and *hellenicum*), which are known marine fish gill pathogens. The analysis included the two MAGs recovered in this family and the closest representative relatives retrieved from NCBI. The values above and below the diagonal show ANI when one genome is queried against the other and vice versa, respectively. The genomes are grouped based on hierarchical clustering applied to the ANI values.
(PDF)

S12 Fig Maximum-likelihood phylogenomic placement of the MAGs in the family Endozoicomonadaceae (order Pseudomonadales). The three MAGs clustered with the genus *Endozoicomonas* – two with *E. atrinae*, which was isolated from the gut of the comb pen shell (*Atrina pectinata*) and one with *E. sp900299555* and *E. elysicola*, fish gill pathogen and a sea slug digestive tract isolate. Support symbols at the nodes represent non-parametric bootstrap values.
(PDF)

S13 Fig Average Nucleotide Identity (ANI) of the MAGs in the family Endozoicomonadaceae (order Pseudomonadales). Two MAGs had a highest ANI of 92% with *Endozoicomonas atrinae*, which was isolated from the gut of the comb pen shell (*Atrina pectinata*), and one had a highest ANI of >96% with *E. sp900299555* and *E. elysicola*, a fish gill pathogen and a sea slug digestive tract isolate. The analysis included the three MAGs recovered in this family and the closest representative relatives retrieved from NCBI. The values above and below the diagonal show ANI when one genome is queried against the other and vice versa, respectively. The genomes were grouped based on hierarchical clustering applied to the ANI values.
(PDF)

S14 Fig Maximum-likelihood phylogenomic placement of the MAGs in the family HTCC2089 (order Pseudomonadales). These MAGs clustered with species in the genus *UBA4421*, in particular with *UBA4421 sp004213835* and *UBA4421 sp002726925* that were isolated from seawater in the Mediterranean Sea and Pacific Ocean, respectively. Support symbols at the nodes represent non-parametric bootstrap values.
(PDF)

S15 Fig Average Nucleotide Identity (ANI) of the MAGs in the family HTCC2089 (order Pseudomonadales). The average ANI between the MAGs and their closest relatives, *UBA4421 sp004213835* and *UBA4421 sp002726925*, was 85%. These relatives were isolated from seawater in the Mediterranean Sea and Pacific Ocean, respectively. The analysis included the three MAGs recovered in this family and the closest representative relatives retrieved from NCBI. The values above and below the diagonal show ANI when one genome is queried against the other and vice versa, respectively. The genomes were grouped based on hierarchical clustering applied to the ANI values.
(PDF)

S16 Fig Maximum-likelihood phylogenomic placement of the three MAGs in the order Rhizobiales. These MAGs (highlighted) form a distinct clade that is sister to the WTKA01 family. Family names are indicated by a leading “f” where family nodes were collapsed. Triangles size represent the number of genomes in these collapsed nodes. The tree is rooted with the order *Caulobacterales* (in red). Support values at the nodes represent non-parametric bootstrap values.
(PDF)

S17 Fig Average Nucleotide Identity (ANI) of the three MAGs in the order Rhizobiales. The MAGs had highest ANI of 69% with *WTKA01 sp011524605* that were isolated from coral reef macroalgae biofilm. The analysis included the three MAGs recovered in this order and the closest representative relatives retrieved from NCBI. The values above and below the diagonal show ANI when one genome is queried against the other and vice versa, respectively. The genomes were grouped based on hierarchical clustering applied to the ANI values.
(PDF)

S18 Fig Maximum-likelihood phylogenomic placement of the five MAGS in the order Flavobacteriales. These MAGS belong to the family Flavobacteriaceae, but to two different clades (general genus *MS024-2A* and *Arcticimaribacter*). These two groups are mainly represented by seawater taxa. Support symbols at the nodes represent non-parametric bootstrap values.
(PDF)

S19 Fig Average Nucleotide Identity (ANI) of the two MAGs in the family Flavobacteriaceae, genus *Arcticimaribacter.* *Arcticimaribacter* is abbreviated as *A.* The MAGs had highest ANI of 78% with *A. sp011526285* that was isolated from coral reef seawater. The analysis included the two MAGs recovered in this genus and the closest representative relatives retrieved from NCBI. The values above and below the diagonal show ANI when one genome is queried against the other and vice versa, respectively. The genomes were grouped based on hierarchical clustering applied to the ANI values.
(PDF)

S20 Fig Average Nucleotide Identity (ANI) of the three MAGs in the family Flavobacteriaceae, genus *MS024-2A.* *MS024-2A* is abbreviated as *M.* The MAGs had an average ANI of 71.3% with *M. sp018699935* that was isolated from hypoxic seawater. The analysis included the three MAGs recovered in this genus and the closest representative relatives retrieved from NCBI. The values above and below the diagonal show ANI when one genome is queried against the other and vice versa, respectively. The genomes were grouped based on hierarchical clustering applied to the ANI values.
(PDF)

S21 FigCompleteness of KEGG metabolic modules in the order Burkholderiales.All MAGs belong to the most prevalent lineage in the Burkholderiaceae family except for MAG23-MAG24 relate to genus *Ichthyocystis* fish gill pathogens in family *2013Ark19i*. The color scale represents module completeness, from 0 to 1. Only modules with completeness ≥75% in at least one MAG are presented. Only MAGs with completeness ≥70% are presented.(PDF)

S22 FigCorrelation between MAG completeness and number of metabolic modules per MAG in the Burkholderiaceae family.Each point represents a metagenome-assembled genome (MAG). The number of metabolic modules was defined as modules with ≥75% completeness. Only MAGs with completeness ≥70% are included.(PDF)

S23 FigCompleteness of KEGG metabolic modules in the genus *Endozoicomonas* (order Pseudomonadales).MAGs 26–27 relate to a species from the gut of the comb pen shell, and MAG29 to fish gill pathogens (but note that it has low completeness). The color scale represents module completeness, from 0 to 1. Only modules with completeness ≥75% in at least one genome are presented.(PDF)

S24 FigCompleteness of KEGG metabolic modules in the genus *UBA4421* (order Pseudomonadales).Three MAGs (30, 31 and 65) were recovered in this genus, which relates to seawater taxa. The color scale represents module completeness, from 0 to 1. Only modules with completeness ≥75% in at least one genome are presented.(PDF)

S25 FigCompleteness of KEGG metabolic modules in the genus *WTKA01* (order Rhizobiales).Three MAGs (44, 45, 67) were recovered in this genus, which relate to a species associated with coral reef macroalgae biofilm. 2SP means this genome is the second species representative (GCA_022968955_1) for the same species according to GTDB. The color scale represents module completeness from 0 to 1. Only modules with completeness ≥75% in at least one genome are presented.(PDF)

S26 FigCompleteness of KEGG metabolic modules in the genus *MS024-2A* (order Flavobacteriales).Three MAGs (57–59) were recovered in this genus, which relate to free-living seawater species. The color scale represents module completeness from 0 to 1. Only modules with completeness ≥75% in at least one genome are presented.(PDF)

S27 FigCompleteness of KEGG metabolic modules in the genus *Arcticimaribacter-2A* (order Flavobacteriales).Two MAGs (55–56) were recovered in this genus, which relate to free-living seawater species. The color scale represents module completeness, from 0 to 1. Only modules with completeness ≥75% in at least one genome are presented.(PDF)

S28 FigCompleteness of KEGG metabolic modules for the five MAGs in the family Flavobacteriaceae.MAG55-MAG56 belong to genus *Arcticimaribacter* while MAG57-MAG59 belong to genus *MS024-2A*. The color scale represent the module completeness from 0 to 1. Only modules with completeness ≥75% in at least one genome are presented.(PDF)

S1 Table Marginal PERMANOVA and interaction models for gill microbiome community composition. PERMANOVA results (adonis2) based on Bray–Curtis dissimilarities calculated from sample-level phylum relative abundance data. The first model presents a marginal PERMANOVA including biogeographic region, host species, sampling location, sampling year, and sequencing run (Seqrun), testing the unique contribution of each factor while controlling for all others. The second and third models test for interactions between fish host species and both sampling year and location, respectively, while accounting for sequencing run and other covariates. The table reports degrees of freedom (Df), sums of squares (SumOfSqs), proportion of variance explained (R2), F-statistic (F) and associated p-values (Pr(>F)) based on 999 permutations. Because biogeographic region is nested within sampling location, its marginal effect was not estimable in some models.
(PDF)

S2 Table Statistical comparison of COG functional category proportions between gill and seawater metagenomes. Mean proportions of each COG category are shown for Bocas 2017 gill and seawater samples. Differences were assessed using two-sample Welch t-tests. P-values were adjusted for multiple testing using the Benjamini–Hochberg false discovery rate (FDR). The column “higher in” indicates the environment with the higher mean proportion.
(PDF)

S3 TableKEGG Ortholog (KO) protein families associated with the Calvin cycle and SOX pathway detected in Burkholderiaceae-B MAGs.Each enzyme is labeled with its KEGG Orthology (KO) accession number. For each enzyme, the table describes its functional annotation (“enzyme definition”), the KEGG module(s) it is associated with (if any), and the number of annotations detected within each MAG. The table lists the number of times each annotation occurs within each MAG. Calvin cycle-associated genes are highlighted in green, whereas SOX pathway genes are highlighted in beige. Only MAGs with ≥70% estimated completeness are included in the table. MAGs highlighted in red represent genomes recovered from single-population assemblies in which all detected Calvin cycle and SOX pathway genes were encoded within the same MAG.(XLSX)

S4 Table KEGG module pathway completeness matrix for all metagenome-assembled genomes (MAGs). Each row represents a KEGG module, while each column corresponds to a MAG. Values in the matrix indicate the module pathwise completeness score, reflecting the proportion of enzymatic steps present in each genome required to complete a given metabolic module. The matrix was generated using anvi-estimate-metabolism.
(XLSX)

S5 Table KEGG Ortholog (KO) protein families annotated in all metagenome-assembled genomes (MAGs). Each enzyme is labeled with its KEGG Orthology (KO) accession number. For each enzyme, the table describes its functional annotation (’enzyme definition’), a comma-separated list of KEGG modules it is associated with (if any), and the number of annotations it has within each MAG. The annotations come from anvi-run-kegg-kofams and the table was exported using anvi-estimate-metabolism. The table lists the number of times each annotation occurs within each MAG.
(XLSX)

S6 Table Functional families of the 2024 release of NCBI COGs annotated across all metagenome-assembled genomes (MAGs). Each functional entry is identified by its accession number and includes its description (“COG24_FUNCTION”). The table lists the number of times each annotation.
(XLSX)

S7 Table List of samples used in this study, including 353 hamlet gill samples, 67 fin clip samples and 7 Serranus gill samples. Date, latitude and longitude refer to collection dates (YYYY-MM-DD) and coordinates. If multiple sequencing runs were available for a sample, all reads were combined.
(XLSX)
