# Supplementary material for: Proteobacteria with chemosynthetic potential are highly prevalent in the gills of Hypoplectrus reef fishes
Source: PLoS Genet. 2026 Aug 28;22(8):e1012266. doi: 10.1371/journal.pgen.1012266 (PMC13552952; doi:10.1371/journal.pgen.1012266)
Supplement: Supplementary file 1 [file pgen.1012266.s001.pdf]

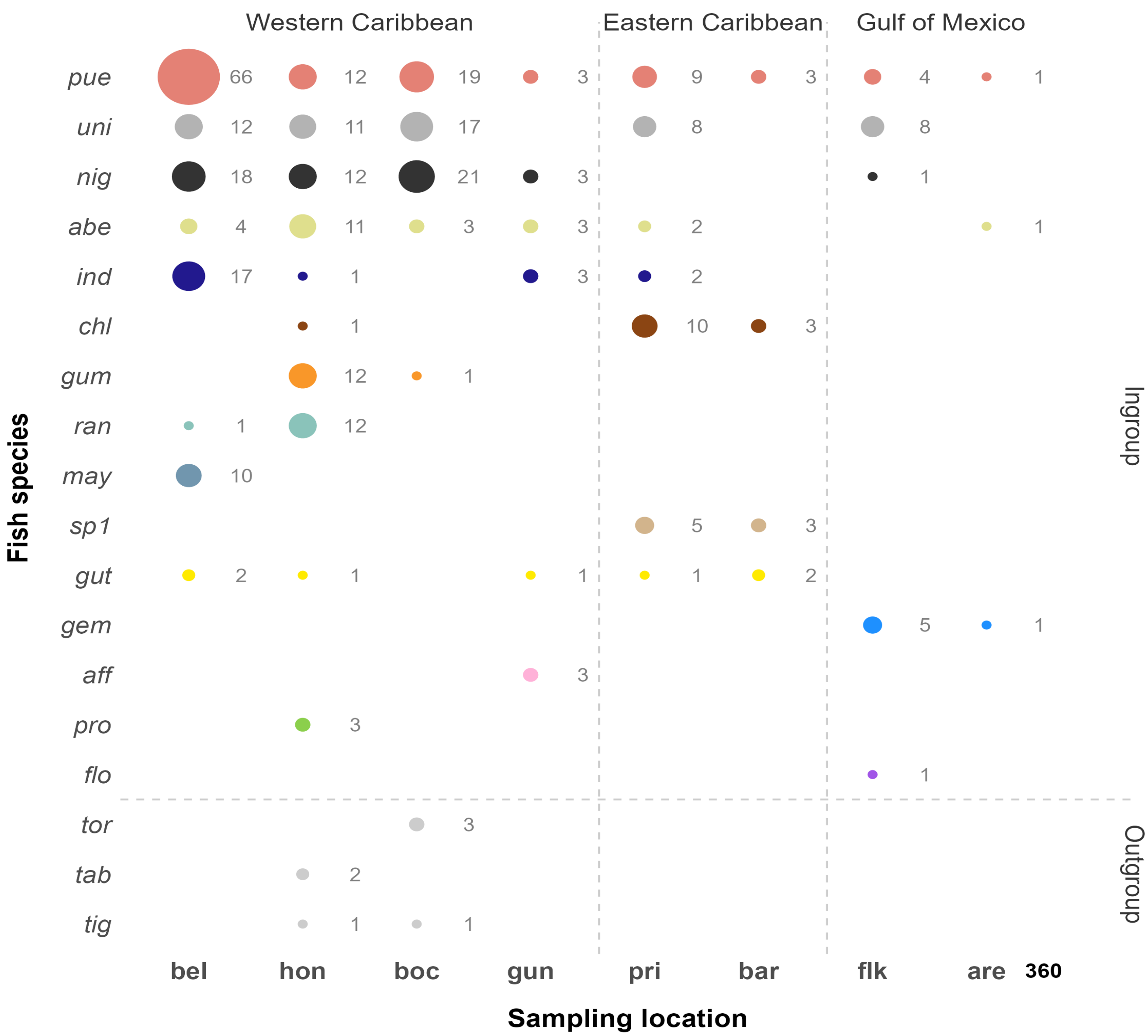

S1 Fig. Overview of the sampling design. The dataset comprises **360 shotgun-sequenced gill samples** representing **18 reef fish species**, including 15 hamlets (*Hypoplectrus*) and 3 outgroup serranids (*Serranus*). Hamlets include pue: *Hypoplectrus puella*, uni: *H. unicolor*, nig: *H. nigricans*, abe: *H. aberrans*, aff: *H. affinis*, chl: *H. chlorurus*, flo: *H. floridae*, gem: *H. gemma*, gum: *H. gummigutta*, gut: *H. guttavarius*, ind: *H. indigo*, may: *H. maya*, pro: *H. providencianus*, ran: *H. randallorum*, and sp1: *Hypoplectrus sp. 1*. Outgroup species comprise tor: *Serranus tortugarum*, tab: *Serranus tabacarius* and tig: *Serranus tigrinus*. Samples were collected at eight locations across the Caribbean – Cayo Arenas (are) and the Florida Keys (flk) in the Gulf of Mexico; Belize (bel), Honduras (hon), Bocas del Toro in Panama (boc) and Guna Yala (gun) in the Western Caribbean; and Puerto Rico (pri) and Barbados (bar) in the Eastern Caribbean.
