# Supplementary material for: Proteobacteria with chemosynthetic potential are highly prevalent in the gills of Hypoplectrus reef fishes
Source: PLoS Genet. 2026 Aug 28;22(8):e1012266. doi: 10.1371/journal.pgen.1012266 (PMC13552952; doi:10.1371/journal.pgen.1012266)
Supplement: Supplementary file 5 [file pgen.1012266.s005.pdf]

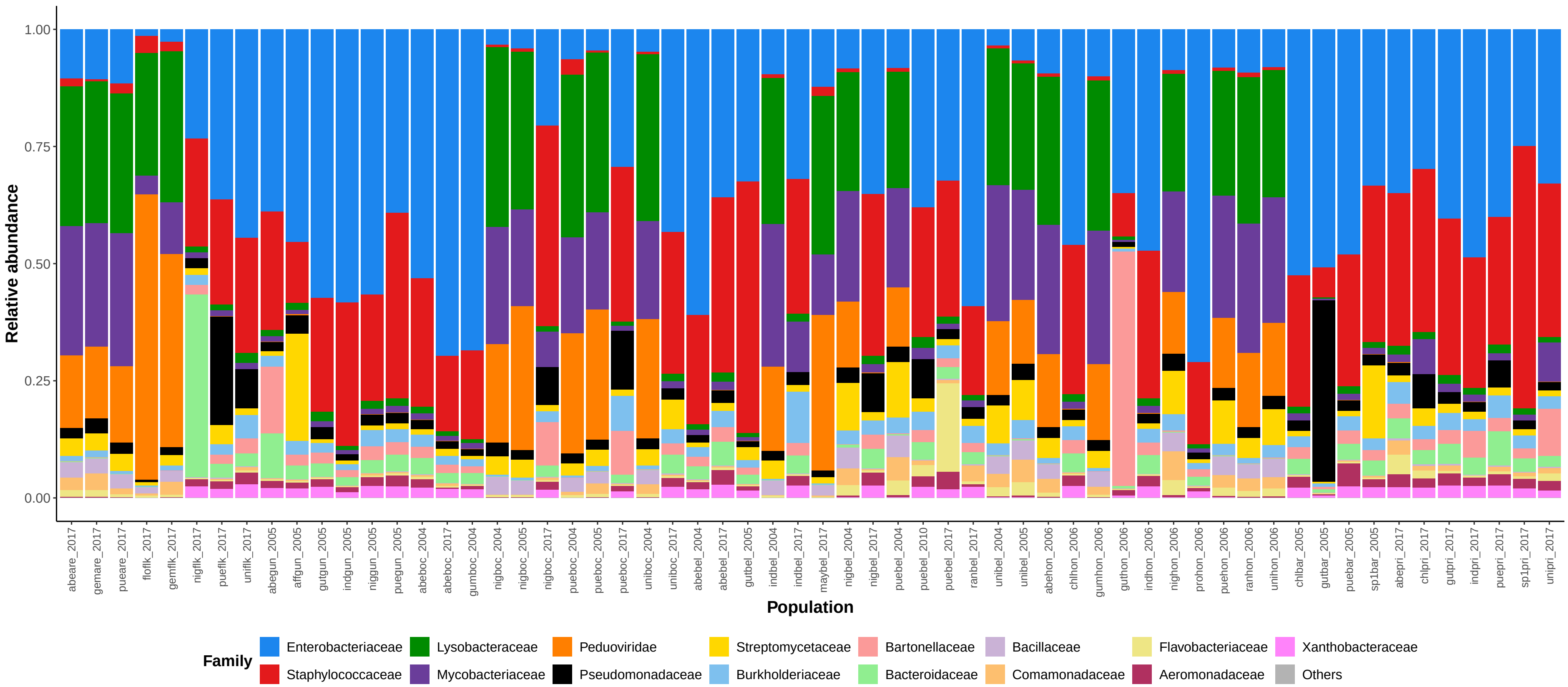

S5 Fig. Taxonomic composition of the gill microbiome at the family level. Each stacked bar represents one of the 60 host populations (fish host species-location-year combination) and shows the mean relative abundance of bacterial phyla averaged across all samples within that population. Taxonomic assignments are based on genomic read classification. The first three letters of the bar labels correspond to the fish host species (pue: *H. puella*, uni: *H. unicolor*, nig: *H. nigricans*, abe: *H. aberrans*, aff: *H. affinis*, chl: *H. chlorurus*, flo: *H. floridae*, gem: *H. gemma*, gum: *H. gummigutta*, gut: *H. guttavarius*, ind: *H. indigo*, may: *H. maya*, pro: *H. providencianus*, ran: *H. randallorum*, sp1: *Hypoplectrus sp. 1*), and the subsequent three letters refer to Caribbean sampling sites: Cayo Arenas (are), Florida Keys (flk), Belize (bel), Honduras (hon), Bocas del Toro, Panama (boc), Guna Yala (gun), Puerto Rico (pri) and Barbados (bar).
