# Supplementary material for: Proteobacteria with chemosynthetic potential are highly prevalent in the gills of Hypoplectrus reef fishes
Source: PLoS Genet. 2026 Aug 28;22(8):e1012266. doi: 10.1371/journal.pgen.1012266 (PMC13552952; doi:10.1371/journal.pgen.1012266)
Supplement: Supplementary file 6 [file pgen.1012266.s006.pdf]

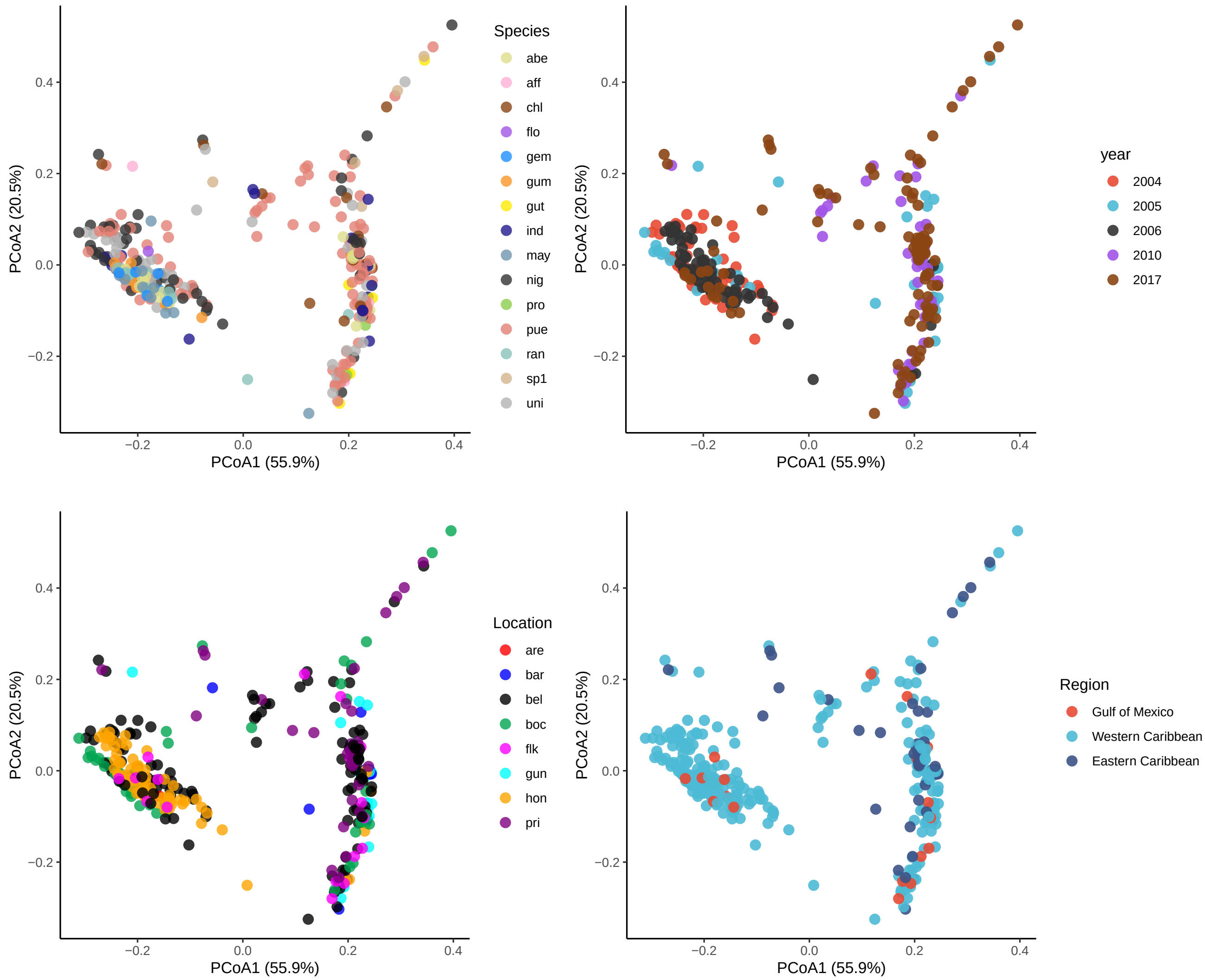

S6 Fig. Principal coordinates analysis (PCoA) of gill microbiome community composition at the phylum level. Ordination was based on Bray–Curtis dissimilarities calculated from sample-level phylum relative abundance data derived from genomic read classification. Samples in ordination are colored according to fish host species (top left), sampling year (top right), sampling location (bottom left) and biogeographic region (bottom right). Fish host species are abbreviated as follows: abe: *H. aberrans*, aff: *H. affinis*, chl: *H. chlorurus*, flo: *H. floridae*, gem: *H. gemma*, gum: *H. gummigutta*, gut: *H. guttavarius*, ind: *H. indigo*, may: *H. maya*, nig: *H. nigricans*, pro: *H. providencianus*, pue: *H. puella*, ran: *H. randallorum*, sp1: *Hypoplectrus sp. 1*, and uni: *H. unicolor*. Sampling locations are abbreviated as follows: Cayo Arenas (are), Barbados (bar), Belize (bel), Bocas del Toro, Panama (boc), Florida Keys (flk), Guna Yala (gun), Honduras (hon) and Puerto Rico (pri). Biogeographic regions correspond to the Gulf of Mexico, Western Caribbean and Eastern Caribbean.
