# Supplementary material for: Proteobacteria with chemosynthetic potential are highly prevalent in the gills of Hypoplectrus reef fishes
Source: PLoS Genet. 2026 Aug 28;22(8):e1012266. doi: 10.1371/journal.pgen.1012266 (PMC13552952; doi:10.1371/journal.pgen.1012266)
Supplement: Supplementary file 7 [file pgen.1012266.s007.pdf]

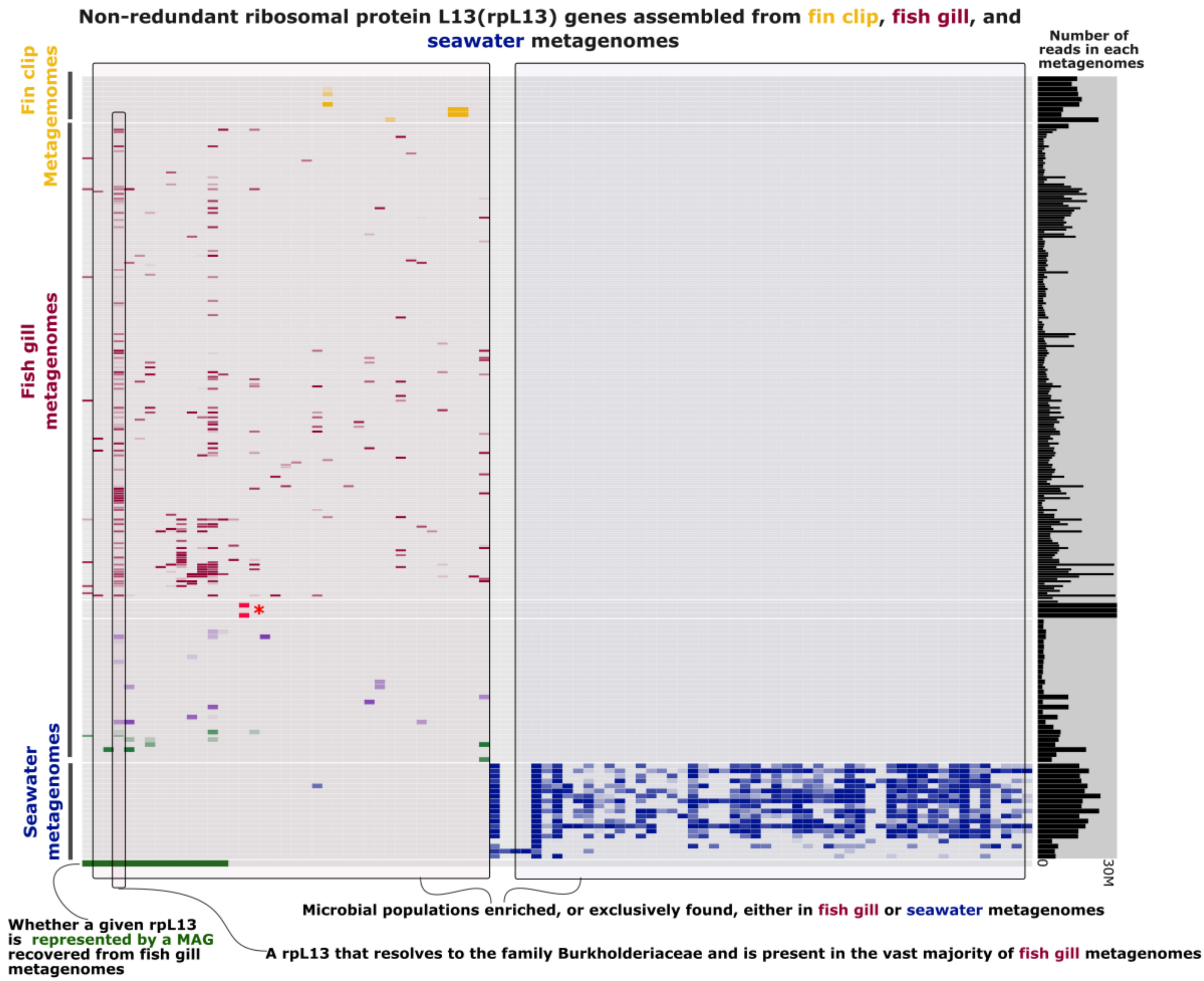

S7 Fig. Ribosomal protein L13 (*rpL13*) genes assembled from fin clip, fish gill and seawater metagenomes. *RPL13* was used as a proxy for the detection of microbial clades. Rows correspond to metagenomic samples, and columns represent *rpL13* representative sequences clustered at 95% nucleotide identity. Heatmap values indicate presence/absence based on read recruitment (70–100% detection). The heatmap highlights differences between gill and seawater samples, including a highly prevalent clade in gills that is absent from seawater, corresponding to a novel lineage within the Burkholderiaceae family. The green part of the horizontal bar at the bottom indicates whether a given rpL13 represented by a MAG recovered from fish gill metagenomes. Red star denotes gill samples from *Serranus* fish. Gill samples collected Bocas 2017 colored in green and gill samples from Bocas 2004/2005 in purple, both just above seawater samples
