# Supplementary material for: Proteobacteria with chemosynthetic potential are highly prevalent in the gills of Hypoplectrus reef fishes
Source: PLoS Genet. 2026 Aug 28;22(8):e1012266. doi: 10.1371/journal.pgen.1012266 (PMC13552952; doi:10.1371/journal.pgen.1012266)
Supplement: Supplementary file 8 [file pgen.1012266.s008.pdf]

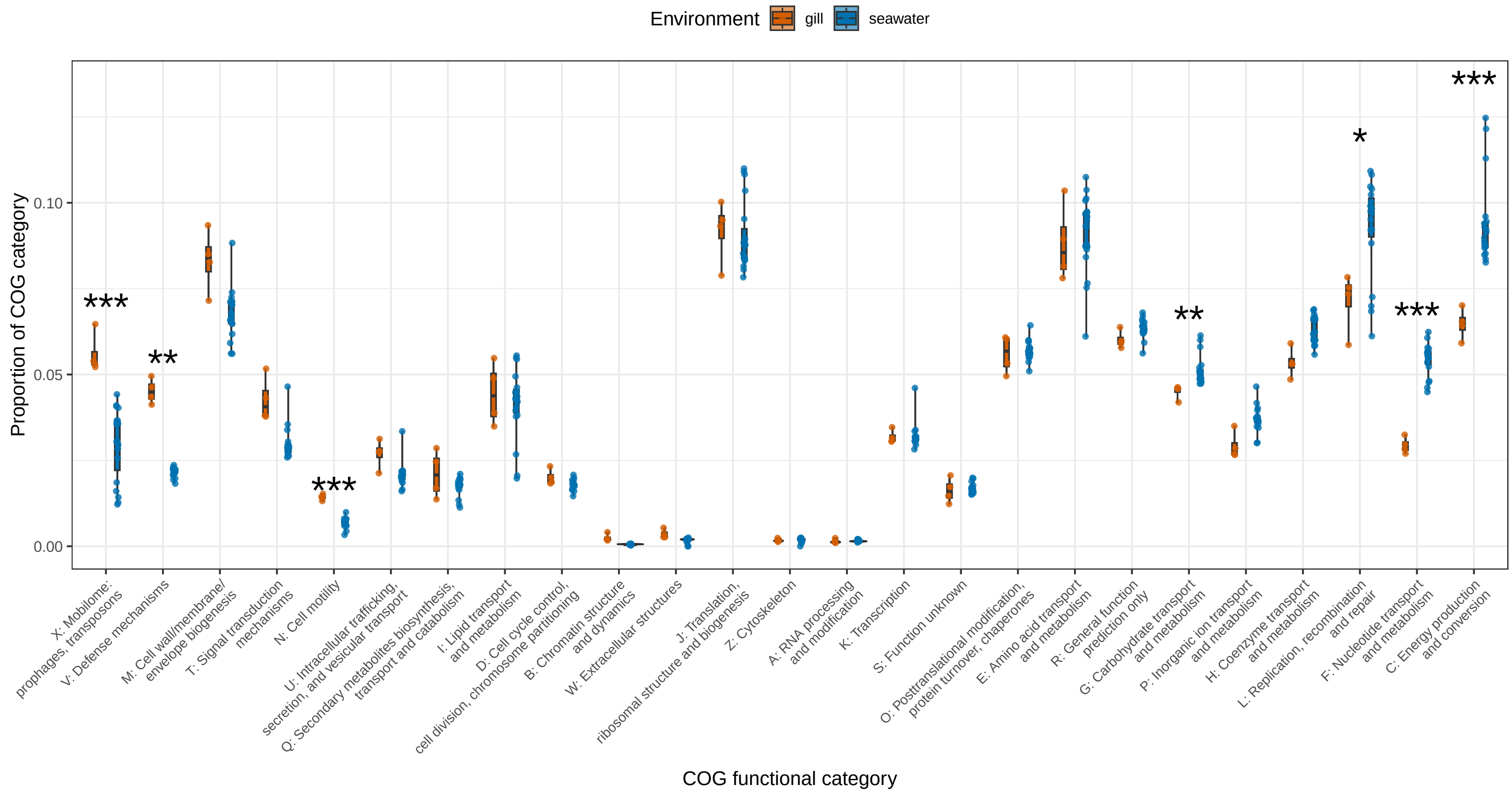

S8 Fig. Comparison of COG functional category proportions between Bocas 2017 gill and seawater metagenomes. Violin plots with overlaid boxplots show the distribution of relative abundances of COG functional categories across individual metagenomes from gill and seawater environments, with points representing individual samples. Functional categories are ordered by the difference in mean proportions between environments (gill minus seawater). Asterisks denote statistically significant differences based on two-sample Welch's t-tests with Benjamini–Hochberg false discovery rate correction (*, $padj < 0.05$; **, $padj < 0.01$; ***, $padj < 0.001$).
