# Supplementary material for: Proteobacteria with chemosynthetic potential are highly prevalent in the gills of Hypoplectrus reef fishes
Source: PLoS Genet. 2026 Aug 28;22(8):e1012266. doi: 10.1371/journal.pgen.1012266 (PMC13552952; doi:10.1371/journal.pgen.1012266)
Supplement: Supplementary file 9 [file pgen.1012266.s009.pdf]

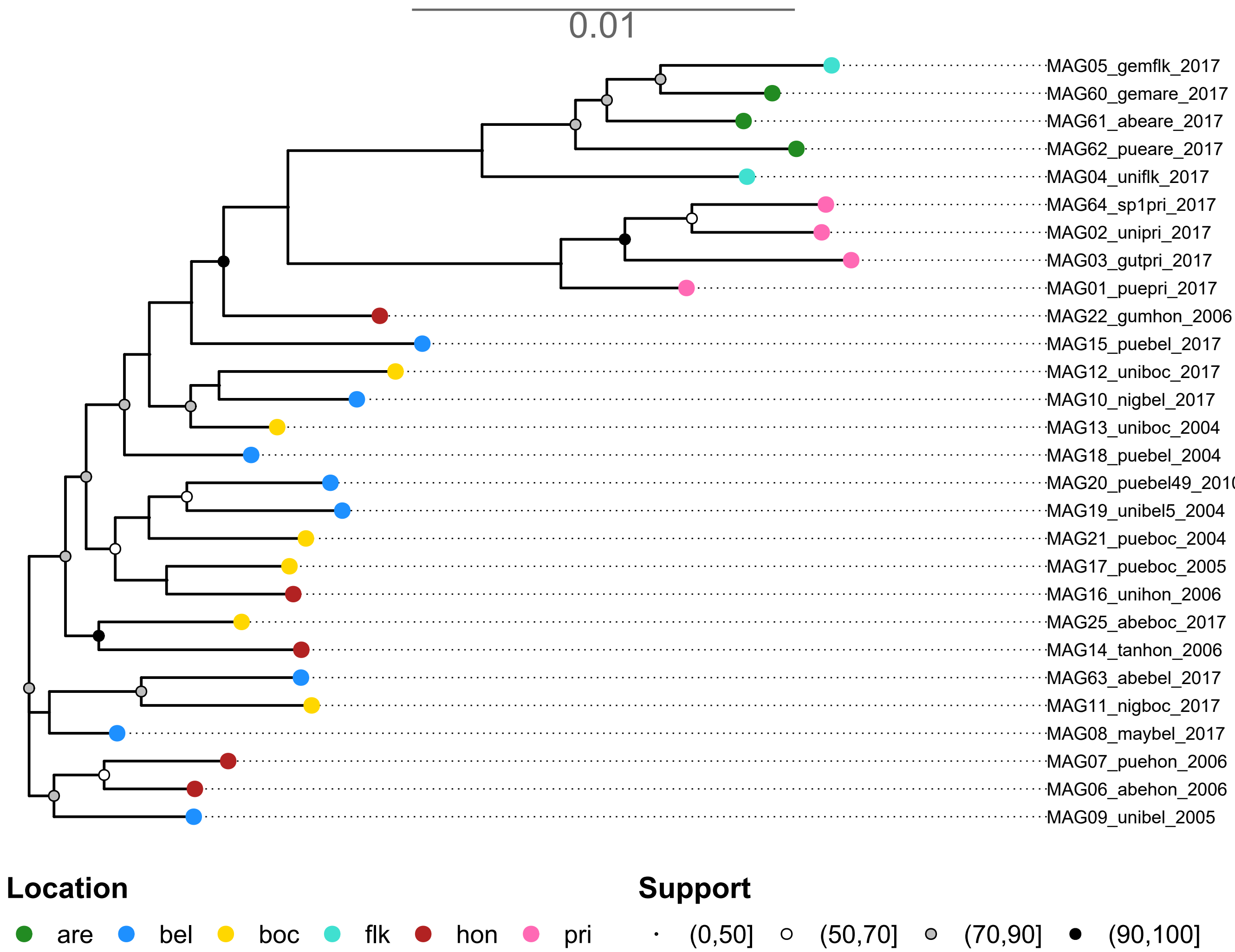

S9 Fig. Maximum-likelihood phylogenetic tree of 28 MAGs from the most prevalent lineage in the Burkholderiaceae family. The MAGs from Cayo Arenas (are) and the Florida Keys (flk) in the Gulf of Mexico and Puerto Rico (pri) formed a clade diverged from the remaining locations: Belize (bel), Honduras (hon) and Bocas del Toro in Panama (boc).
