# Supplementary material for: Proteobacteria with chemosynthetic potential are highly prevalent in the gills of Hypoplectrus reef fishes
Source: PLoS Genet. 2026 Aug 28;22(8):e1012266. doi: 10.1371/journal.pgen.1012266 (PMC13552952; doi:10.1371/journal.pgen.1012266)
Supplement: Supplementary file 10 [file pgen.1012266.s010.pdf]

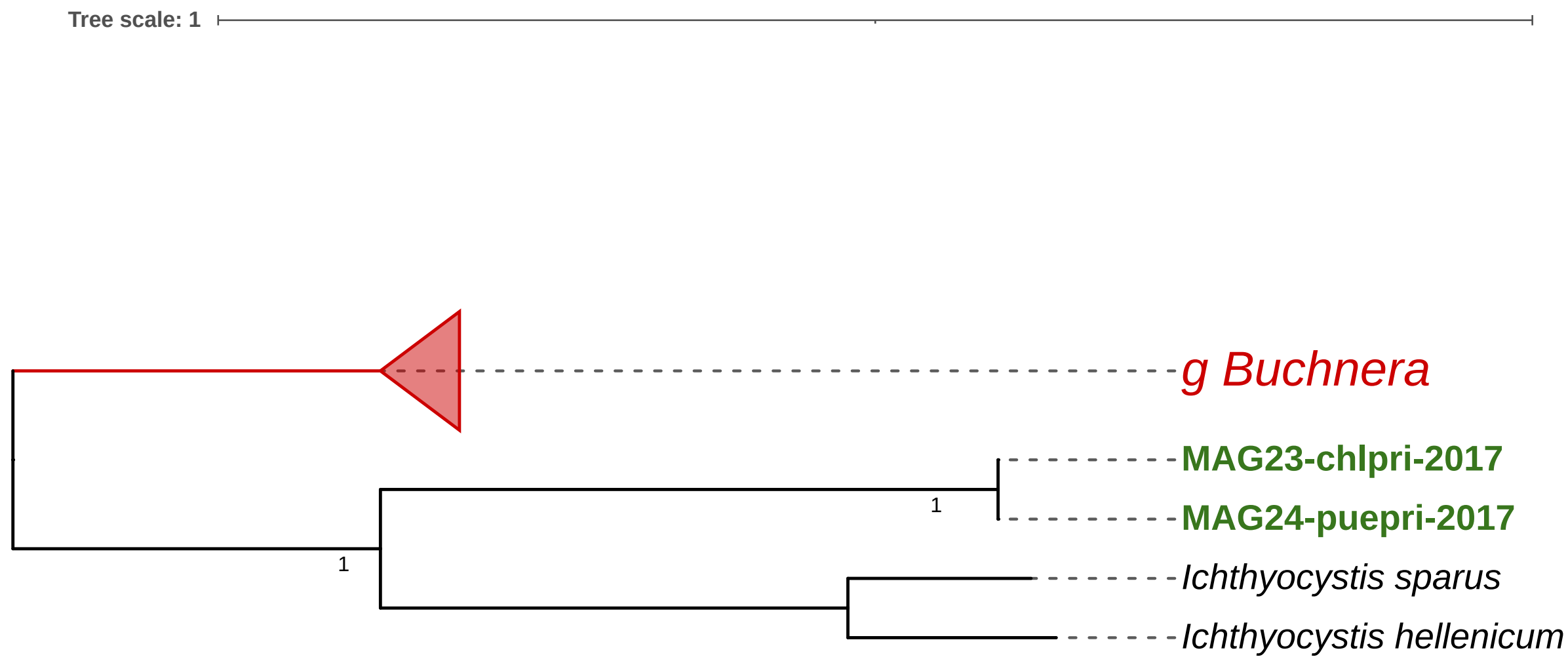

S10 Fig. Maximum-likelihood phylogentic placement of the MAGs in the 2013Ark19i family (order Burkholderiales). The tree is rooted with the genus *Buchnera* (in red). Support values at the nodes represent non-parametric bootstrap values.
