# Supplementary material for: Proteobacteria with chemosynthetic potential are highly prevalent in the gills of Hypoplectrus reef fishes
Source: PLoS Genet. 2026 Aug 28;22(8):e1012266. doi: 10.1371/journal.pgen.1012266 (PMC13552952; doi:10.1371/journal.pgen.1012266)
Supplement: Supplementary file 11 [file pgen.1012266.s011.pdf]

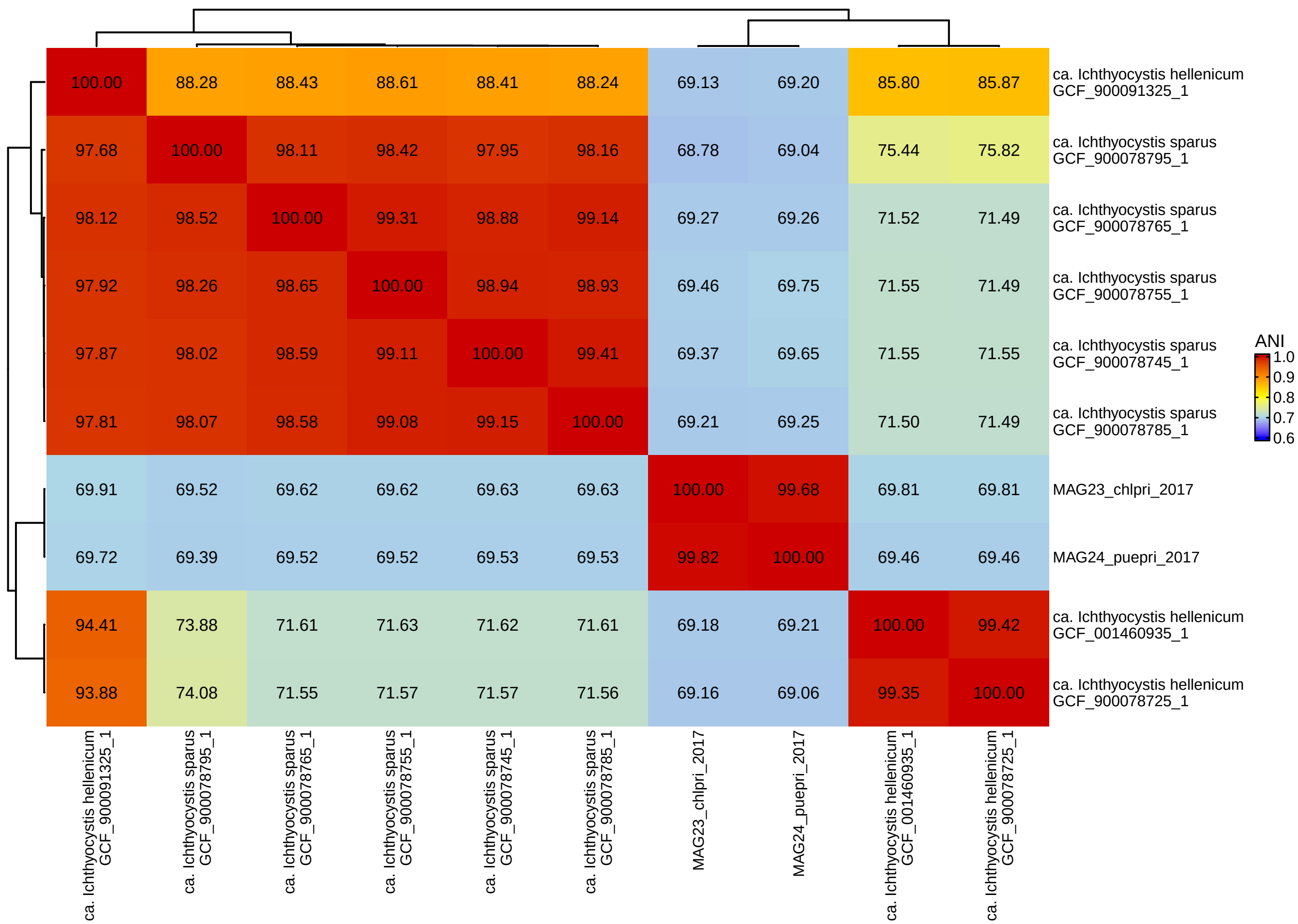

S11 Fig. Average Nucleotide Identity (ANI) of the MAGs in the 2013Ark19i family (order Burkholderiales). These two MAGs were very closely related (> 99% ANI) but showed an ANI < 70% with the other genomes in the family (*Ichthyocystis sparus* and *hellenicum*), which are known marine fish gill pathogens. The analysis included the two MAGs recovered in this family and the closest representative relatives retrieved from NCBI. The values above and below the diagonal show ANI when one genome is queried against the other and vice-versa, respectively. The genomes are grouped based on hierarchical clustering applied to the ANI values.
