# Supplementary material for: Proteobacteria with chemosynthetic potential are highly prevalent in the gills of Hypoplectrus reef fishes
Source: PLoS Genet. 2026 Aug 28;22(8):e1012266. doi: 10.1371/journal.pgen.1012266 (PMC13552952; doi:10.1371/journal.pgen.1012266)
Supplement: Supplementary file 12 [file pgen.1012266.s012.pdf]

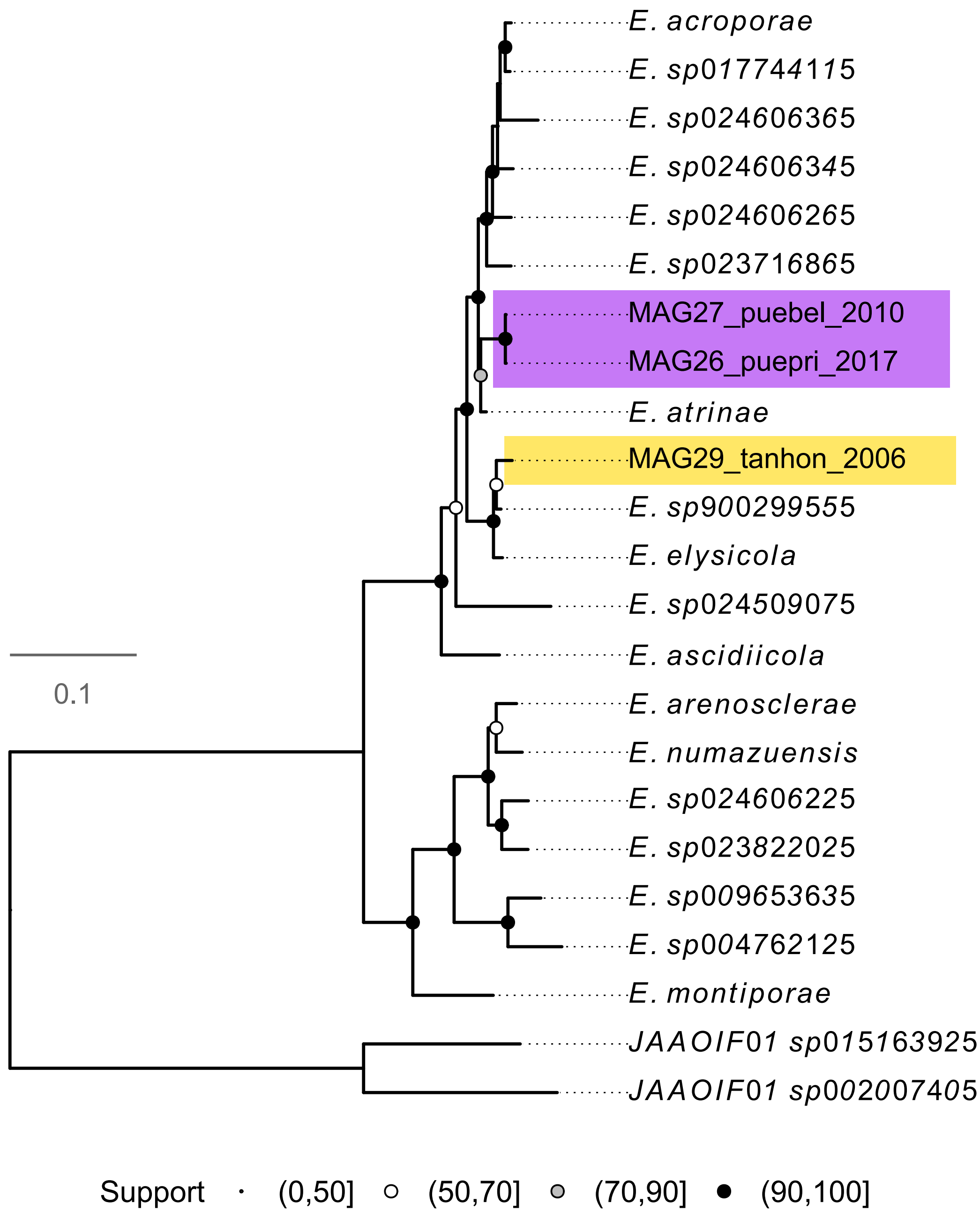

S12 Fig. Maximum-likelihood phylogenomic placement of the MAGs in the family Endozoicomonadaceae (order Pseudomonadales). The three MAGs clustered with the genus *Endozoicomonas* – two with *E. atrinae*, which was isolated from the gut of the comb pen shell (*Atrina pectinata*) and one with *E. sp900299555* and *E. elysicola*, fish gill pathogen and a sea slug digestive tract isolate. Support symbols at the nodes represent non-parametric bootstrap values.
