# Supplementary material for: Proteobacteria with chemosynthetic potential are highly prevalent in the gills of Hypoplectrus reef fishes
Source: PLoS Genet. 2026 Aug 28;22(8):e1012266. doi: 10.1371/journal.pgen.1012266 (PMC13552952; doi:10.1371/journal.pgen.1012266)
Supplement: Supplementary file 13 [file pgen.1012266.s013.pdf]

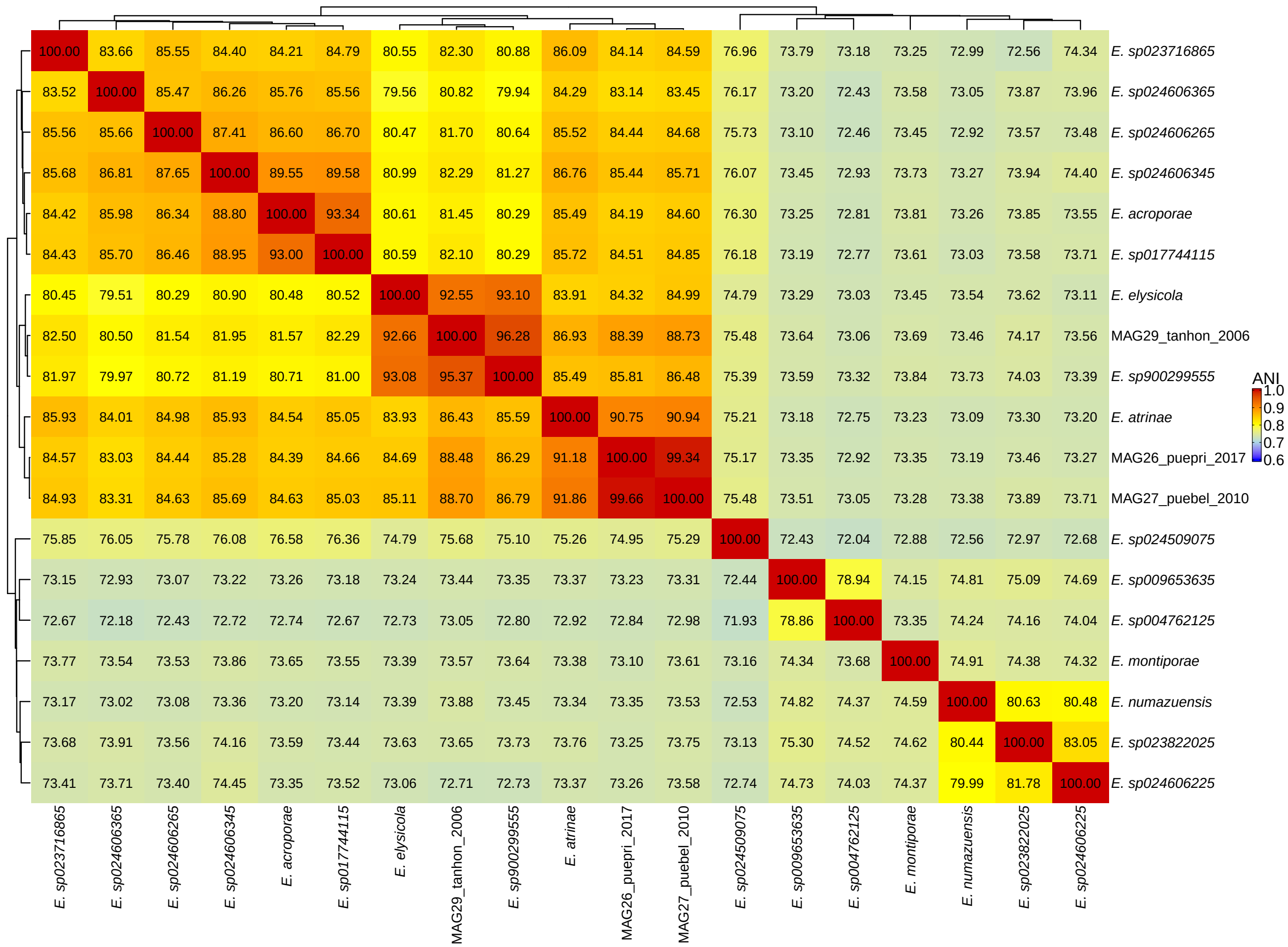

S13 Fig. Average Nucleotide Identity (ANI) of the MAGs in the family Endozoicomonadaceae (order Pseudomonadales). Two MAGs had a highest ANI of 92% with *Endozoicomonas atrinae*, which was isolated from the gut of the comb pen shell (*Atrina pectinata*), and one had a highest ANI of > 96% with *E. sp900299555* and *E. elysicola*, a fish gill pathogen and a sea slug digestive tract isolate, respectively. The analysis included the three MAGs recovered in this family and the closest representative relatives retrieved from NCBI. The values above and below the diagonal show ANI when one genome is queried against the other and vice versa, respectively. The genomes were grouped based on hierarchical clustering applied to the ANI values.
