# Supplementary material for: Proteobacteria with chemosynthetic potential are highly prevalent in the gills of Hypoplectrus reef fishes
Source: PLoS Genet. 2026 Aug 28;22(8):e1012266. doi: 10.1371/journal.pgen.1012266 (PMC13552952; doi:10.1371/journal.pgen.1012266)
Supplement: Supplementary file 14 [file pgen.1012266.s014.pdf]

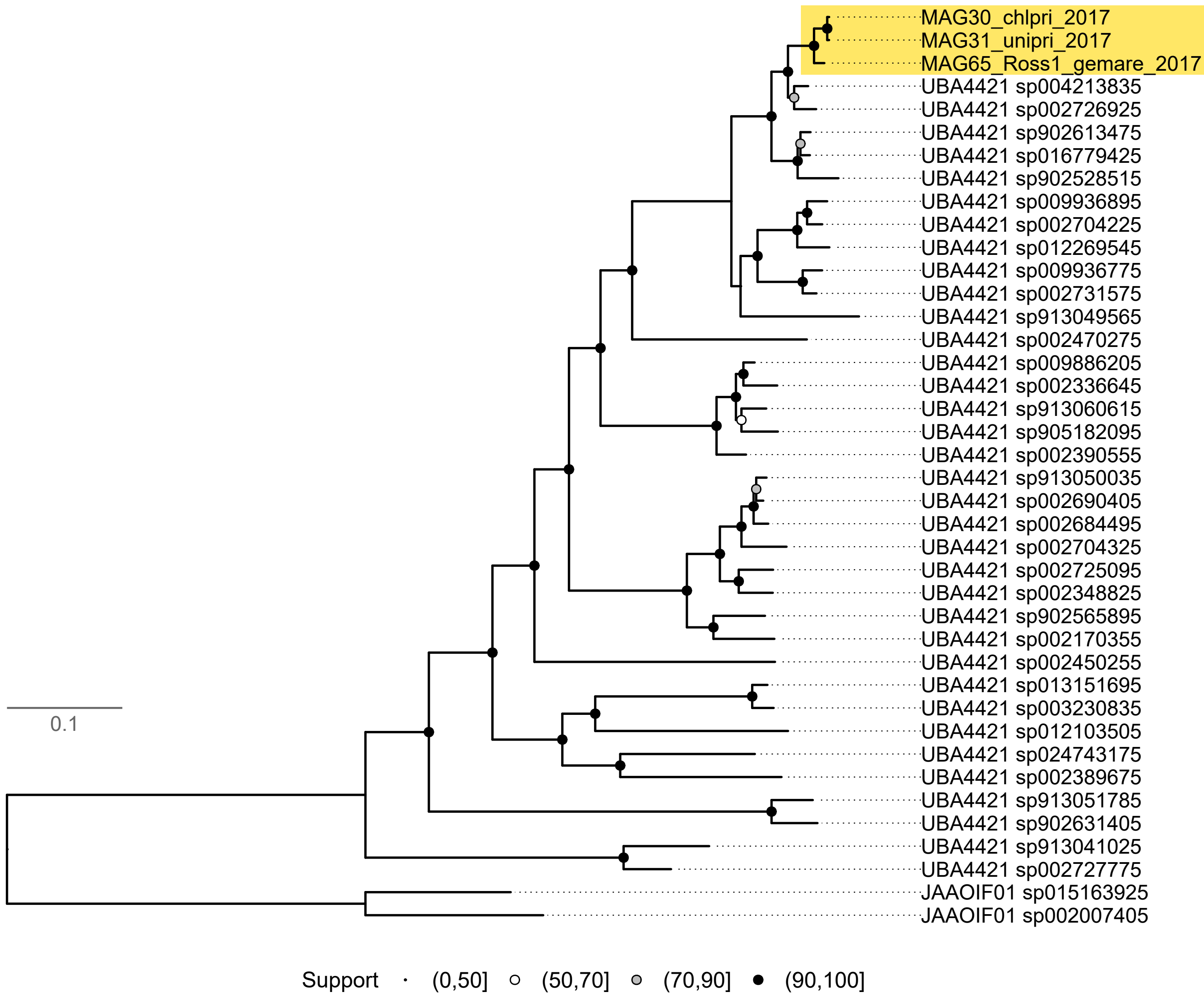

S14 Fig. Maximum-likelihood phylogenomic placement of the MAGs in the family HTCC2089 (order Pseudomonadales). These MAGs clustered with species in the genus *UBA4421*, in particular with *UBA4421 sp004213835* and *UBA4421 sp*002726925 that were isolated from seawater in the Mediterranean Sea and Pacific Ocean, respectively. Support symbols at the nodes represent non-parametric bootstrap values.
