# Supplementary material for: Proteobacteria with chemosynthetic potential are highly prevalent in the gills of Hypoplectrus reef fishes
Source: PLoS Genet. 2026 Aug 28;22(8):e1012266. doi: 10.1371/journal.pgen.1012266 (PMC13552952; doi:10.1371/journal.pgen.1012266)
Supplement: Supplementary file 15 [file pgen.1012266.s015.pdf]

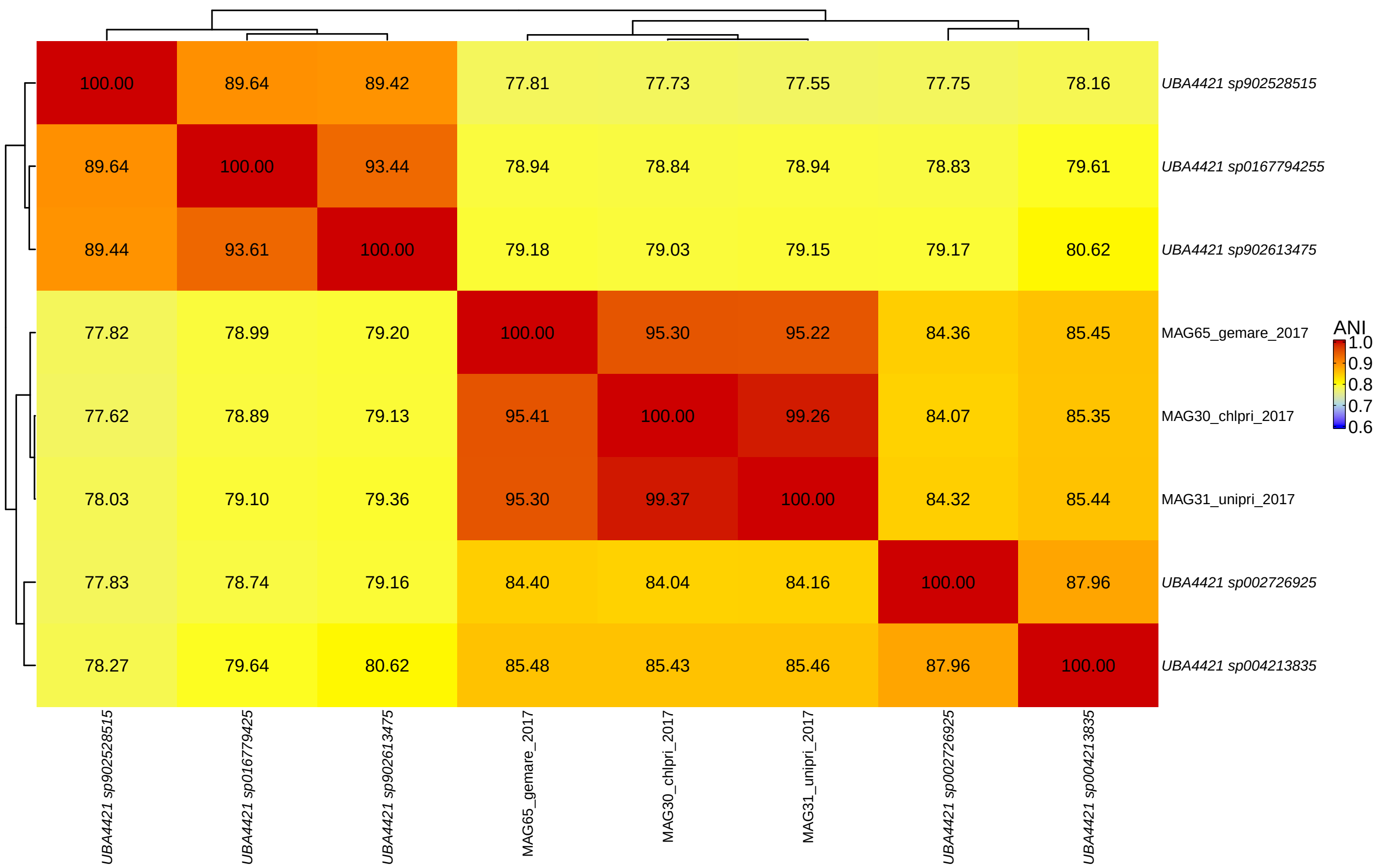

S15 Fig. Average Nucleotide Identity (ANI) of the MAGs in the family HTCC2089 (order Pseudomonadales). The average ANI between the MAGs and their closest relatives, *UBA4421 sp004213835* and *UBA4421 sp002726925*, was 85%. These relatives were isolated from seawater in the Mediterranean Sea and Pacific Ocean, respectively. The analysis included the three MAGs recovered in this family and the closest representative relatives retrieved from NCBI. The values above and below the diagonal show ANI when one genome is queried against the other and vice-versa, respectively. The genomes were grouped based on hierarchical clustering applied to the ANI values.
