# Supplementary material for: Proteobacteria with chemosynthetic potential are highly prevalent in the gills of Hypoplectrus reef fishes
Source: PLoS Genet. 2026 Aug 28;22(8):e1012266. doi: 10.1371/journal.pgen.1012266 (PMC13552952; doi:10.1371/journal.pgen.1012266)
Supplement: Supplementary file 16 [file pgen.1012266.s016.pdf]

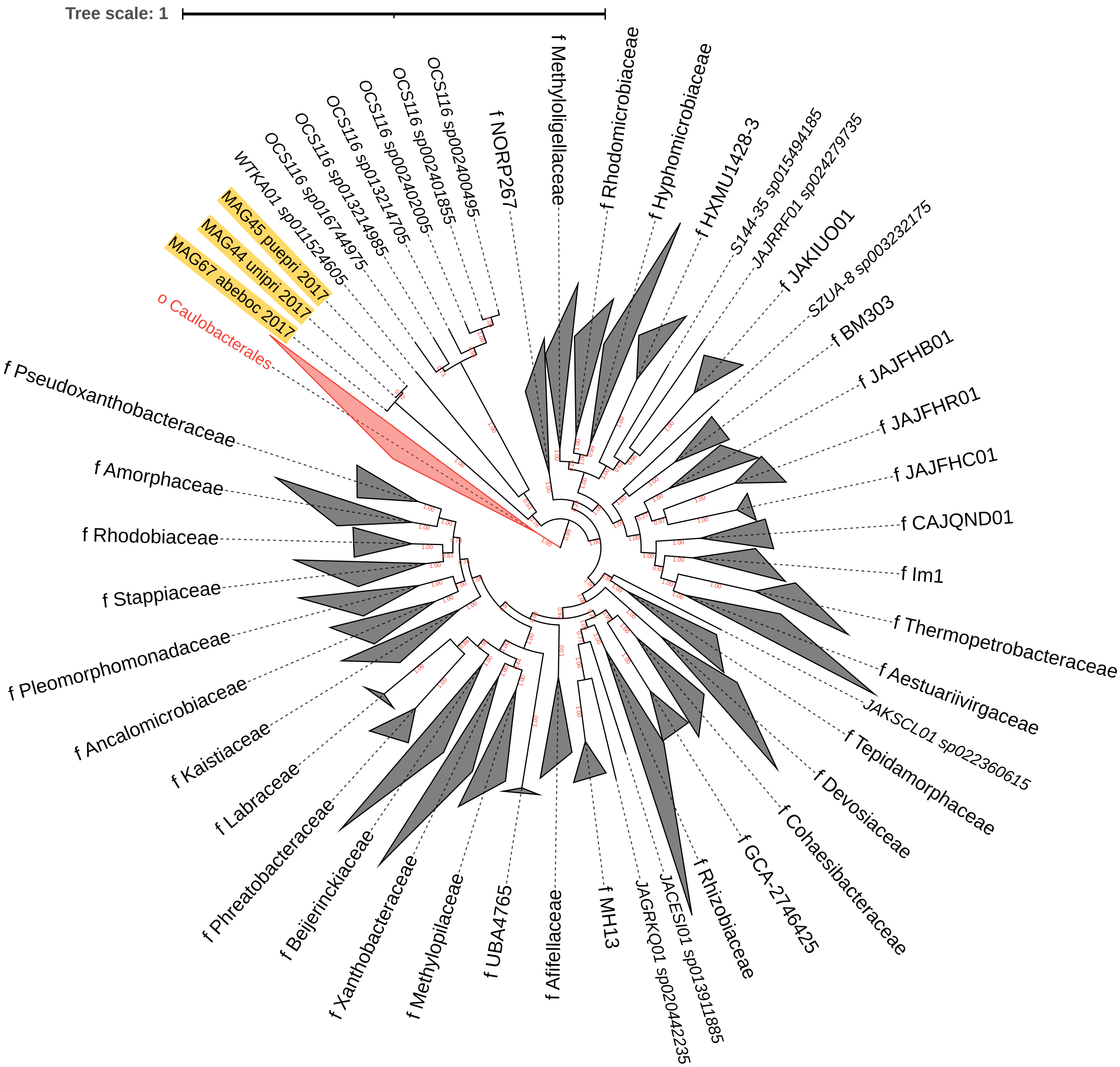

S16 Fig. Maximum-likelihood phylogenomic placement of the three MAGs in the order Rhizobiales. These MAGs (highlighted) form a distinct clade that is sister to the WTKA01 family. Family names are indicated by a leading ”f” where family nodes were collapsed. Triangles size represent the number of genomes in these collapsed nodes. The tree is rooted with the order Caulobacterales (in red). Support values at the nodes represent non-parametric bootstrap values.
