# Supplementary material for: Proteobacteria with chemosynthetic potential are highly prevalent in the gills of Hypoplectrus reef fishes
Source: PLoS Genet. 2026 Aug 28;22(8):e1012266. doi: 10.1371/journal.pgen.1012266 (PMC13552952; doi:10.1371/journal.pgen.1012266)
Supplement: Supplementary file 17 [file pgen.1012266.s017.pdf]

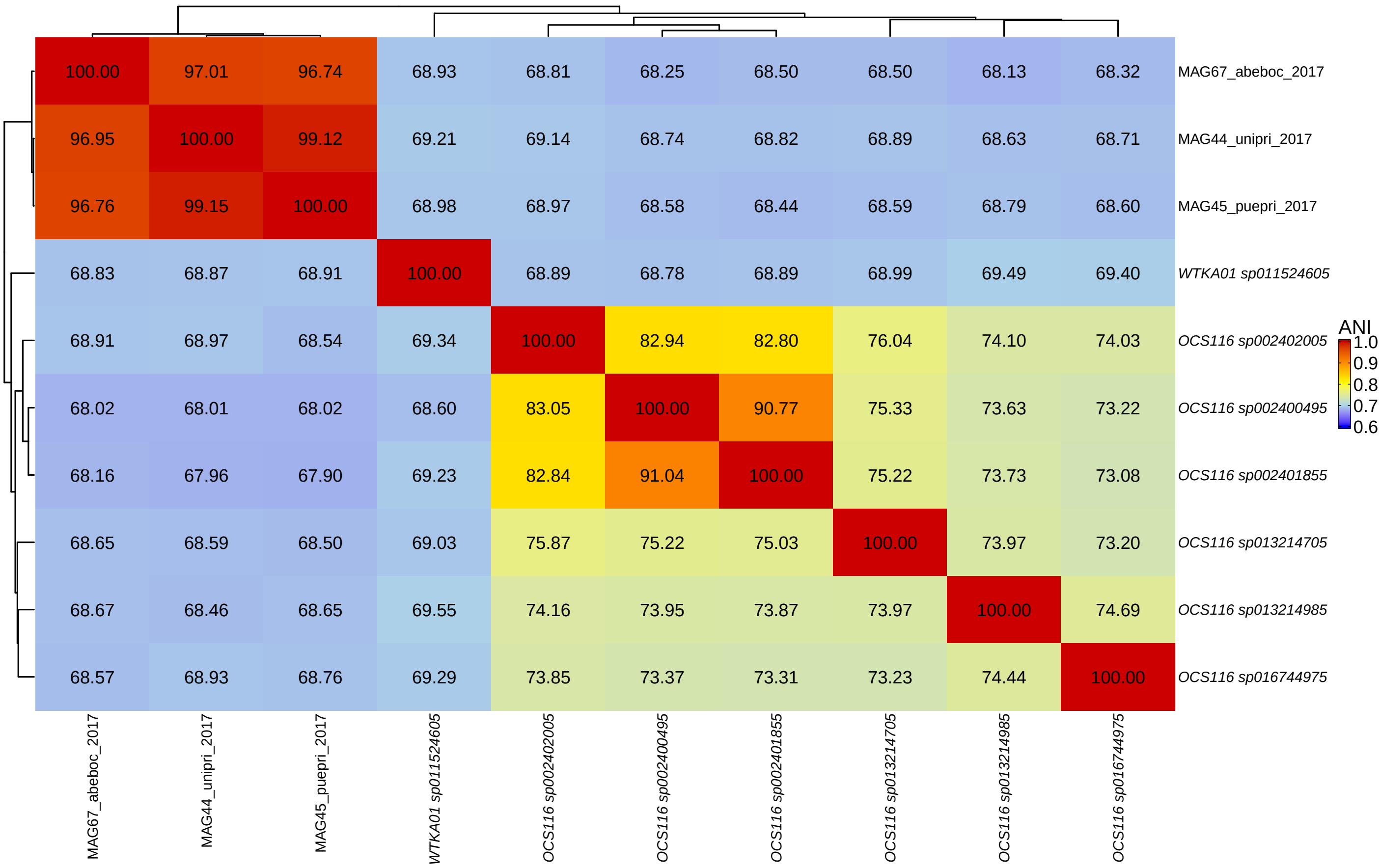

S17 Fig. Average Nucleotide Identity (ANI) of the three MAGs in the order Rhizobiales. The MAGs had highest ANI of 69% with *WTKA01 sp011524605* that were isolated from coral reef macroalgae biofilm. The analysis included the three MAGs recovered in this order and the closest representative relatives retrieved from NCBI. The values above and below the diagonal show ANI when one genome is queried against the other and vice-versa, respectively. The genomes were grouped based on hierarchical clustering applied to the ANI values.
