# Supplementary material for: Proteobacteria with chemosynthetic potential are highly prevalent in the gills of Hypoplectrus reef fishes
Source: PLoS Genet. 2026 Aug 28;22(8):e1012266. doi: 10.1371/journal.pgen.1012266 (PMC13552952; doi:10.1371/journal.pgen.1012266)
Supplement: Supplementary file 18 [file pgen.1012266.s018.pdf]

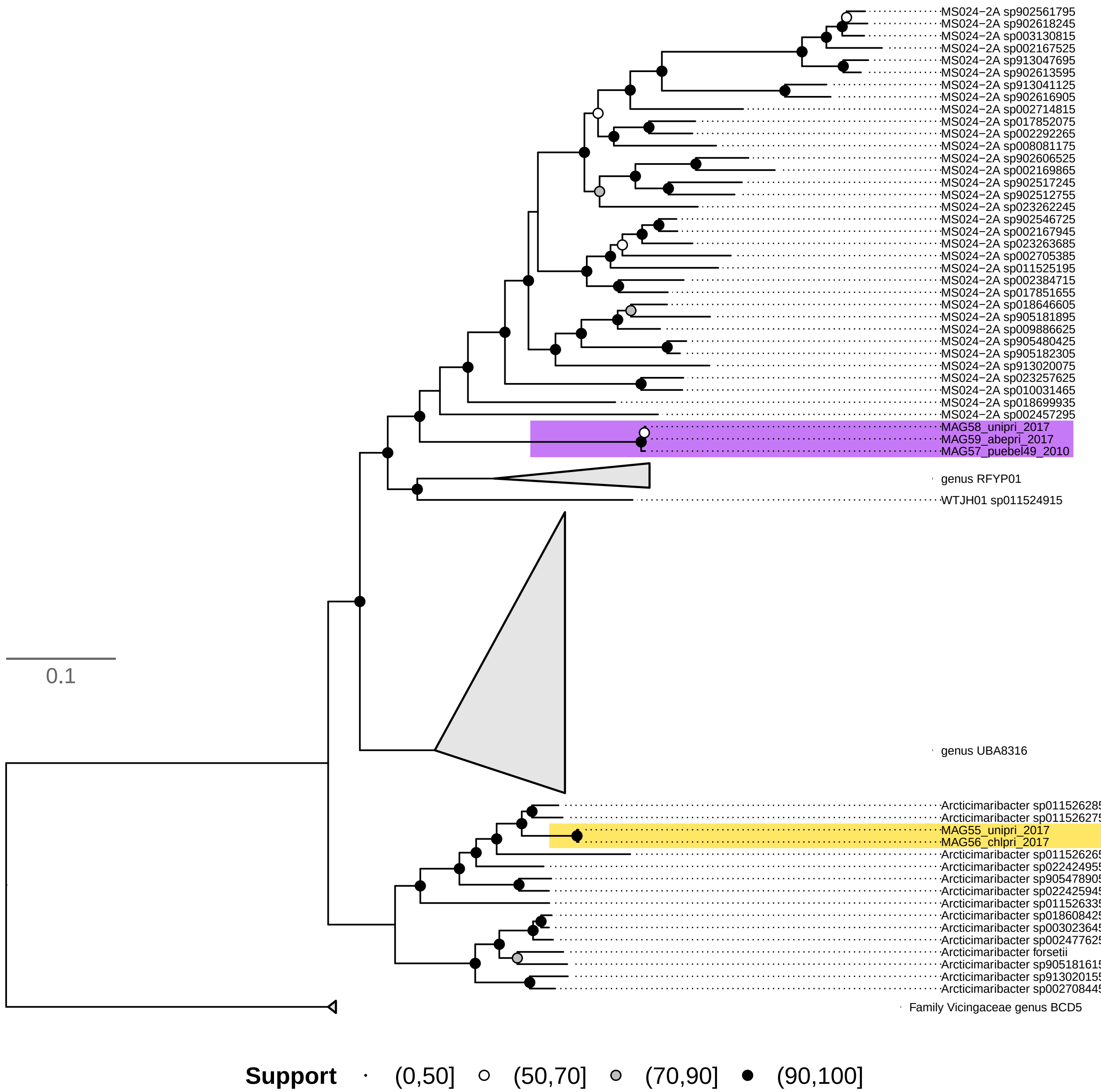

S18 Fig. Maximum-likelihood phylogenomic placement of the five MAGs in the order Flavobacteriales. These MAGs belong to the family Flavobacteriaceae but fall into two distinct clades corresponding to the genera *MS024-2A* and *Arcticimaribacter*. These two clades are mainly represented by seawater-associated taxa. Support symbols at the nodes represent non-parametric bootstrap values.
