# Supplementary material for: Proteobacteria with chemosynthetic potential are highly prevalent in the gills of Hypoplectrus reef fishes
Source: PLoS Genet. 2026 Aug 28;22(8):e1012266. doi: 10.1371/journal.pgen.1012266 (PMC13552952; doi:10.1371/journal.pgen.1012266)
Supplement: Supplementary file 19 [file pgen.1012266.s019.pdf]

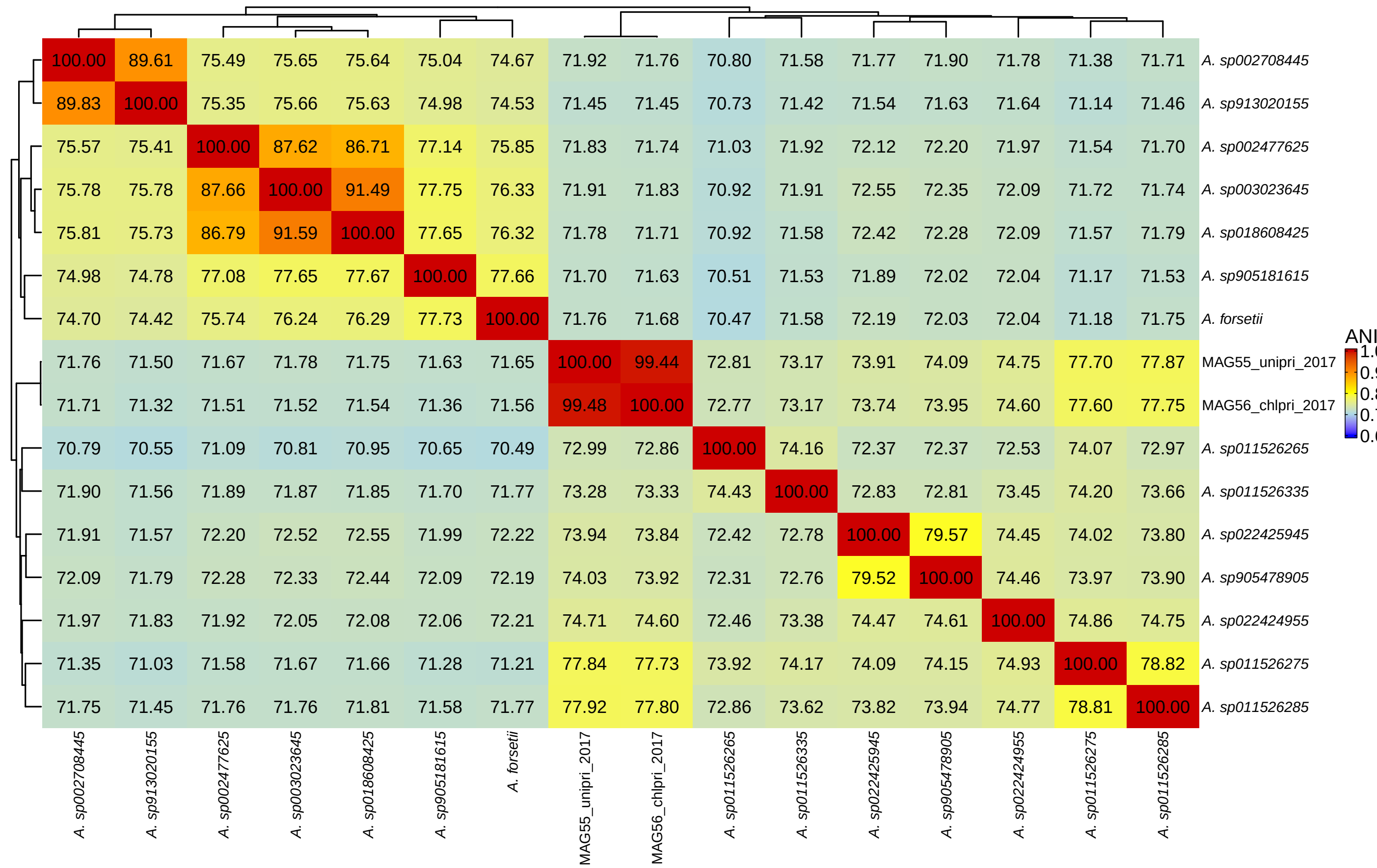

S19 Fig. Average Nucleotide Identity (ANI) of the two MAGs in the family Flavobacteriaceae, genus *Arcticimaribacter*. *Arcticimaribacter* is abbreviated as *A*. The MAGs had the highest ANI (78%) with *A. sp011526285*, that was isolated from coral reef seawater. The analysis included the two MAGs recovered in this genus and the closest representative relatives retrieved from NCBI. The values above and below the diagonal show ANI when one genome is queried against the other and vice versa, respectively. The genomes were grouped based on hierarchical clustering applied to the ANI values.
