# Supplementary material for: Proteobacteria with chemosynthetic potential are highly prevalent in the gills of Hypoplectrus reef fishes
Source: PLoS Genet. 2026 Aug 28;22(8):e1012266. doi: 10.1371/journal.pgen.1012266 (PMC13552952; doi:10.1371/journal.pgen.1012266)
Supplement: Supplementary file 20 [file pgen.1012266.s020.pdf]

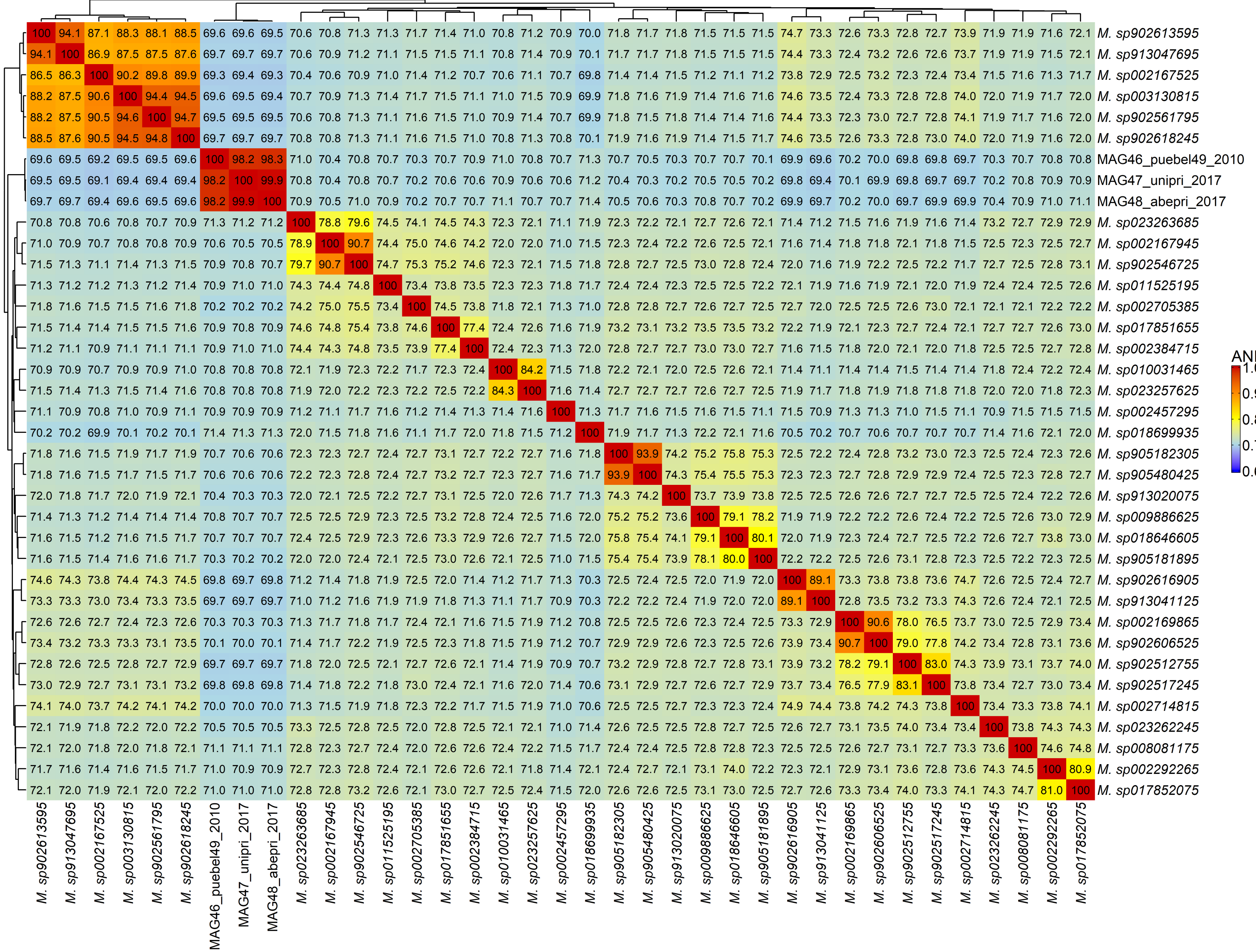


**S20 Fig. Average Nucleotide Identity (ANI) of the three MAGs in the family Flavobacteriaceae, genus *MS024-2A*.** *MS024-2A* is abbreviated as *M*. The MAGs had an average ANI of 71.3% with *M. sp018699935* that was isolated from hypoxic seawater. The analysis included the three MAGs recovered in this genus and the closest representative relatives retrieved from NCBI. The values above and below the diagonal show ANI when one genome is queried against the other and vice versa, respectively. The genomes were grouped based on hierarchical clustering applied to the ANI values.
