# Supplementary material for: Proteobacteria with chemosynthetic potential are highly prevalent in the gills of Hypoplectrus reef fishes
Source: PLoS Genet. 2026 Aug 28;22(8):e1012266. doi: 10.1371/journal.pgen.1012266 (PMC13552952; doi:10.1371/journal.pgen.1012266)
Supplement: Supplementary file 21 [file pgen.1012266.s021.pdf]

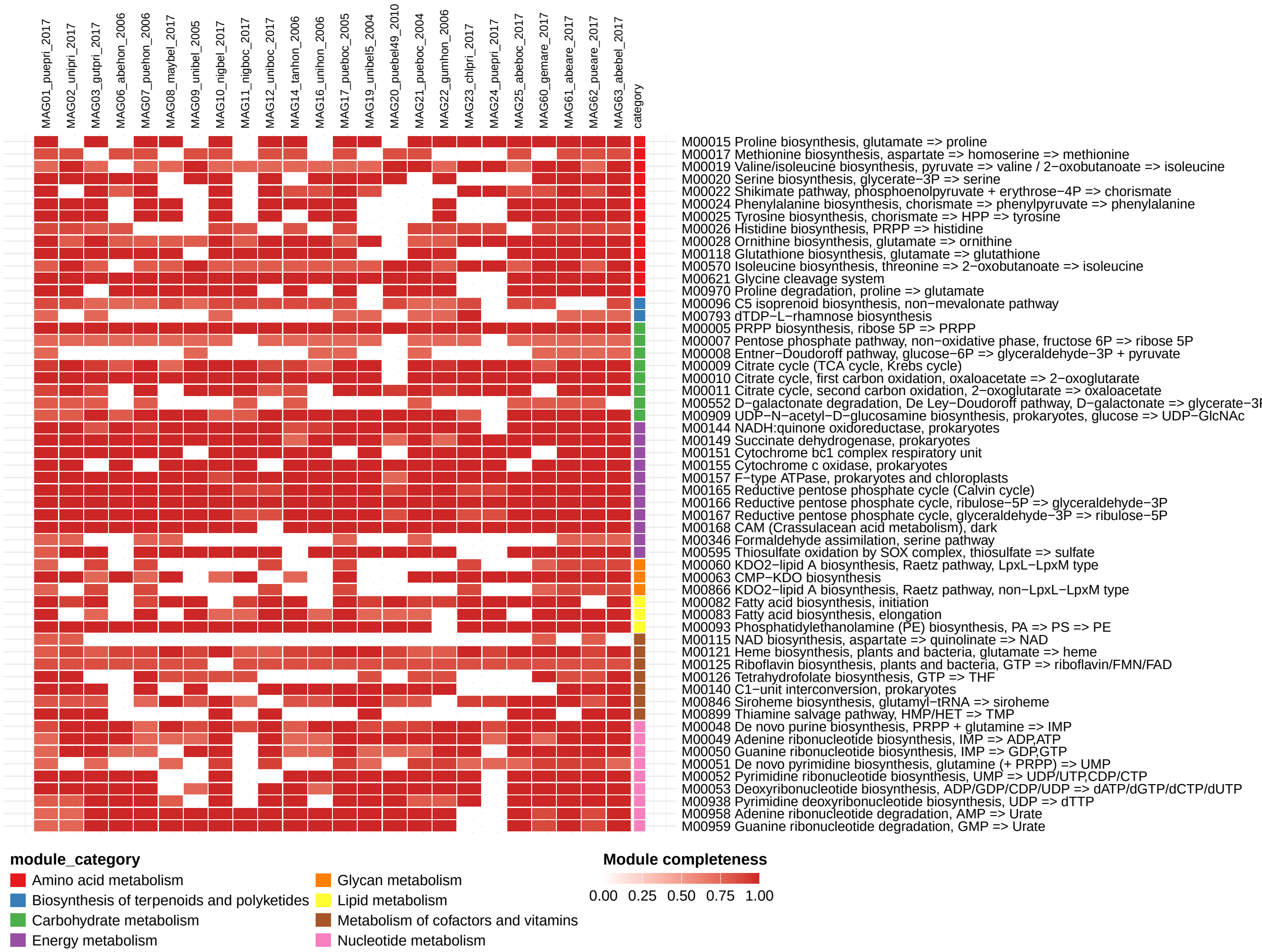

S21 Fig. Completeness of KEGG metabolic modules in the order Burkholderiales. All MAGs belong to the most prevalent lineage in the Burkholderiaceae family except for MAG23 and MAG24, which relate to genus *Ichthyocystis*, a genus that includes known fish gill pathogens in the family 2013Ark19i. The color scale represents module completeness, ranging from 0 to 1. Only modules with completeness $\geq$ 75% in at least one MAG are presented. Only MAGs with completeness $\geq$ 70% are presented.
