# Supplementary material for: Proteobacteria with chemosynthetic potential are highly prevalent in the gills of Hypoplectrus reef fishes
Source: PLoS Genet. 2026 Aug 28;22(8):e1012266. doi: 10.1371/journal.pgen.1012266 (PMC13552952; doi:10.1371/journal.pgen.1012266)
Supplement: Supplementary file 22 [file pgen.1012266.s022.pdf]

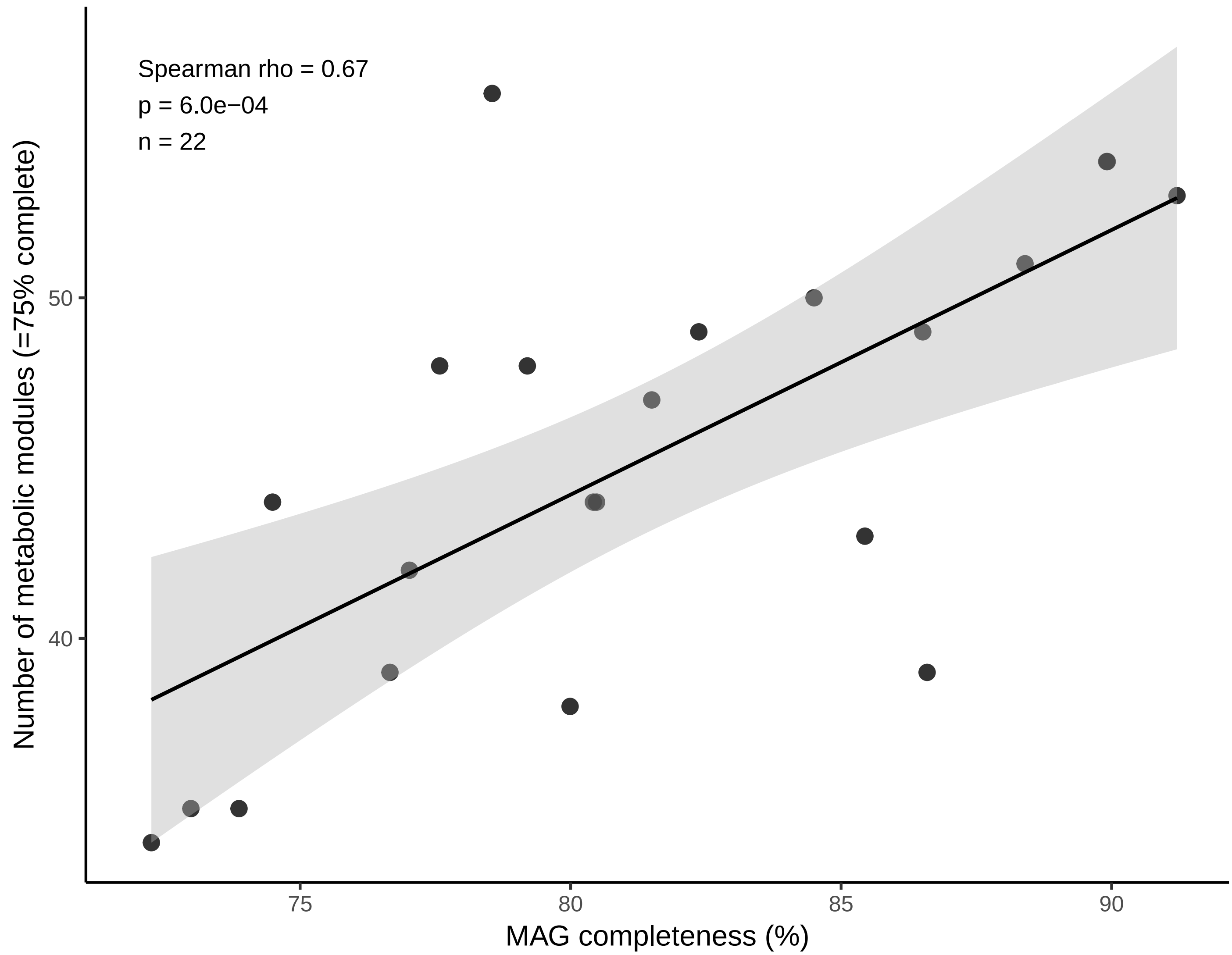

S22 Fig. Correlation between MAG completeness and number of metabolic modules per MAG in the Burkholderiaceae family. Each point represents a metagenome-assembled genome (MAG). The number of metabolic modules was defined as modules with ≥75% completeness. Only MAGs with completeness ≥ 70% are included.
