# Supplementary material for: Proteobacteria with chemosynthetic potential are highly prevalent in the gills of Hypoplectrus reef fishes
Source: PLoS Genet. 2026 Aug 28;22(8):e1012266. doi: 10.1371/journal.pgen.1012266 (PMC13552952; doi:10.1371/journal.pgen.1012266)
Supplement: Supplementary file 23 [file pgen.1012266.s023.pdf]

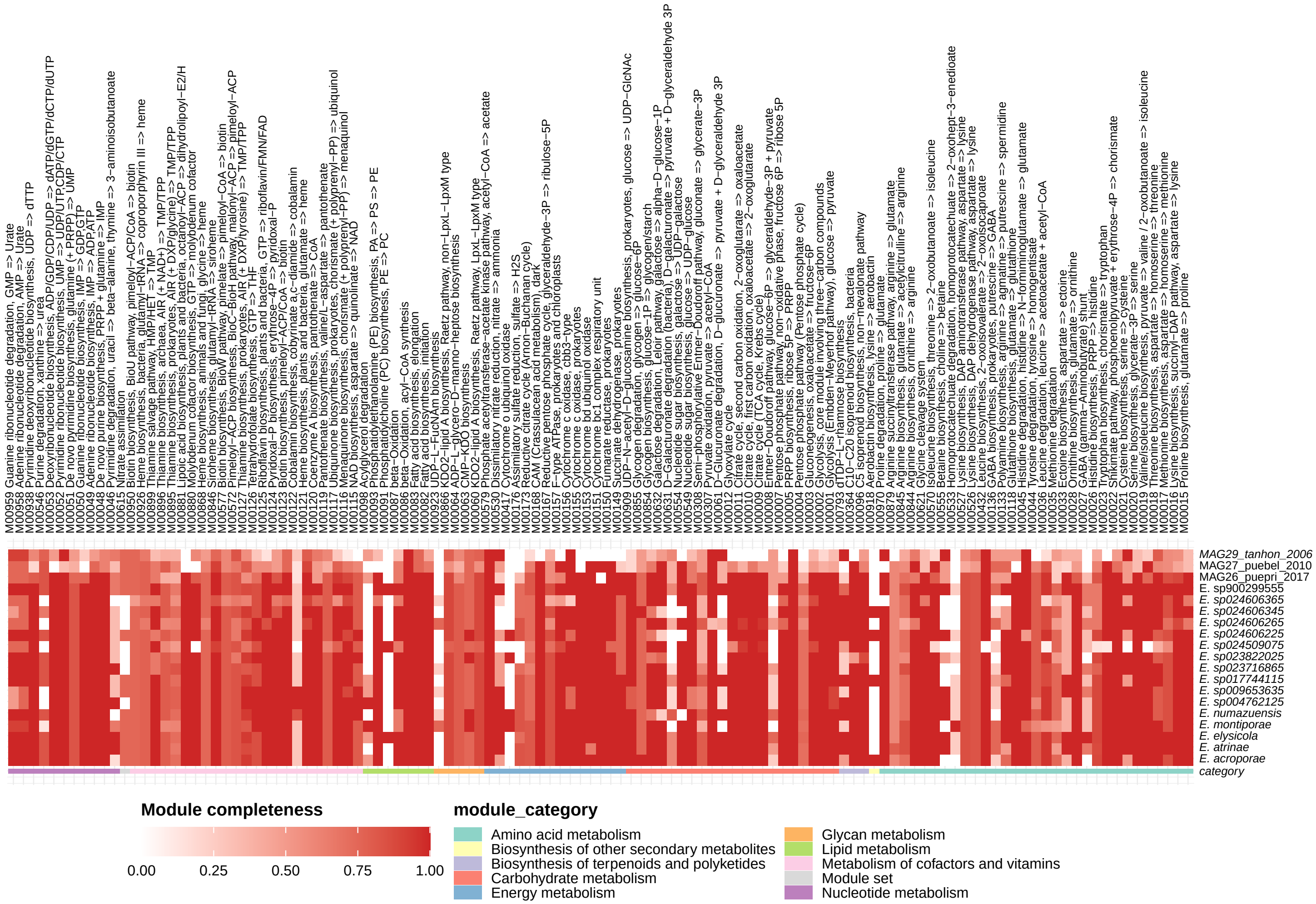

S23 Fig. Completeness of KEGG metabolic modules in the genus *Endozoicomonas* (order Pseudomonadales). MAGs 26-27 relate to a species from the gut of the comb pen shell, and MAG29 to fish gill pathogens (but note that it has low completeness). The color scale represents module completeness, ranging from 0 to 1. Only modules with completeness ≥ 75% in at least one genome are presented.
