# Supplementary material for: Proteobacteria with chemosynthetic potential are highly prevalent in the gills of Hypoplectrus reef fishes
Source: PLoS Genet. 2026 Aug 28;22(8):e1012266. doi: 10.1371/journal.pgen.1012266 (PMC13552952; doi:10.1371/journal.pgen.1012266)
Supplement: Supplementary file 25 [file pgen.1012266.s025.pdf]

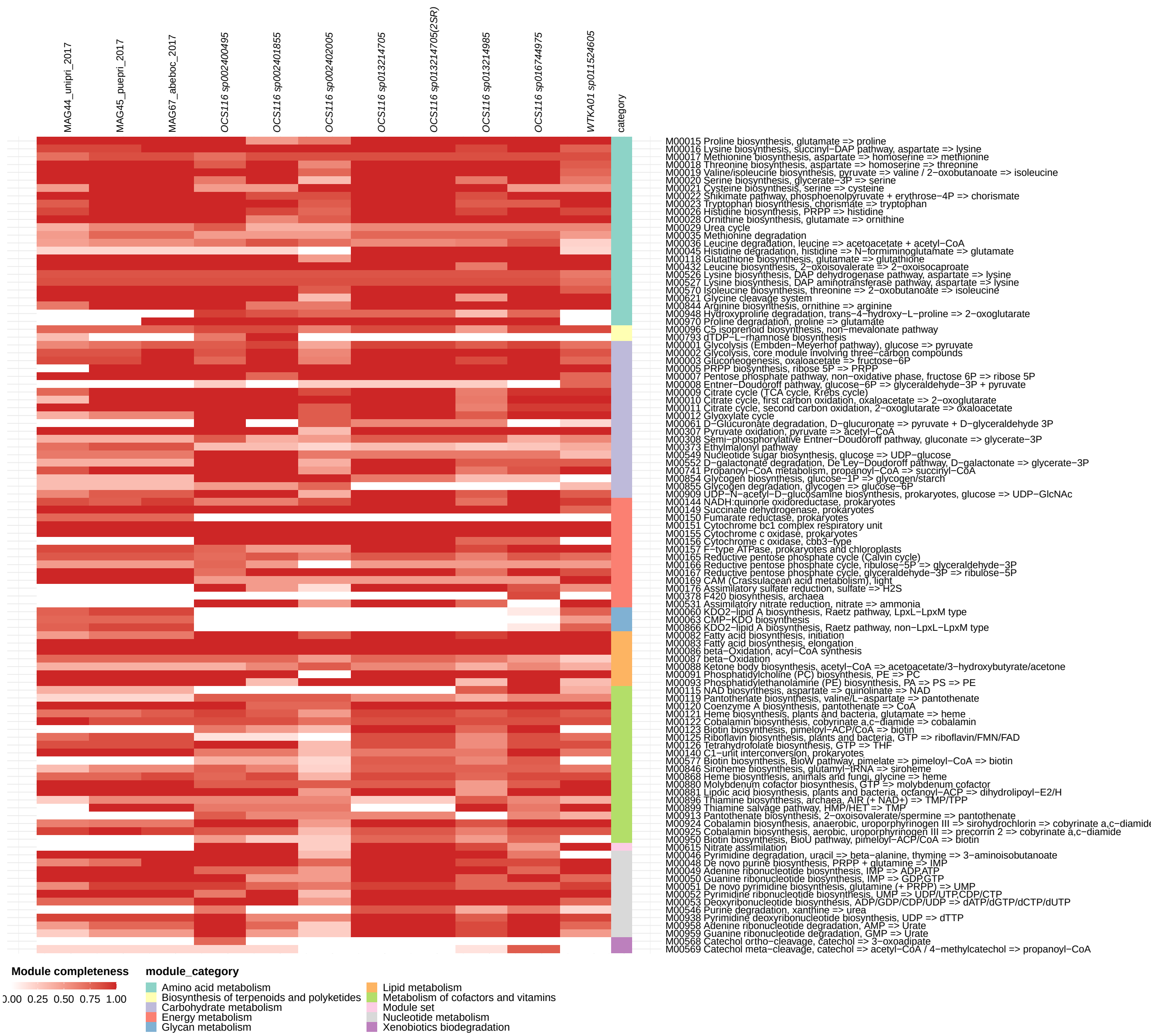

S25 Fig. Completeness of KEGG metabolic modules in the genus *WTKA01* (order Rhizobiales). Three MAGs (44, 45 and 67) were recovered in this genus, which relate to a species associated with coral reef macroalgal biofilm. 2SP means this genome is the second species representative (GCA_022968955_1) for the same species according to GTDB. The color scale represents module completeness ranging from 0 to 1. Only modules with completeness ≥ 75% in at least one genome are presented.
