# Supplementary material for: Proteobacteria with chemosynthetic potential are highly prevalent in the gills of Hypoplectrus reef fishes
Source: PLoS Genet. 2026 Aug 28;22(8):e1012266. doi: 10.1371/journal.pgen.1012266 (PMC13552952; doi:10.1371/journal.pgen.1012266)
Supplement: Supplementary file 26 [file pgen.1012266.s026.pdf]

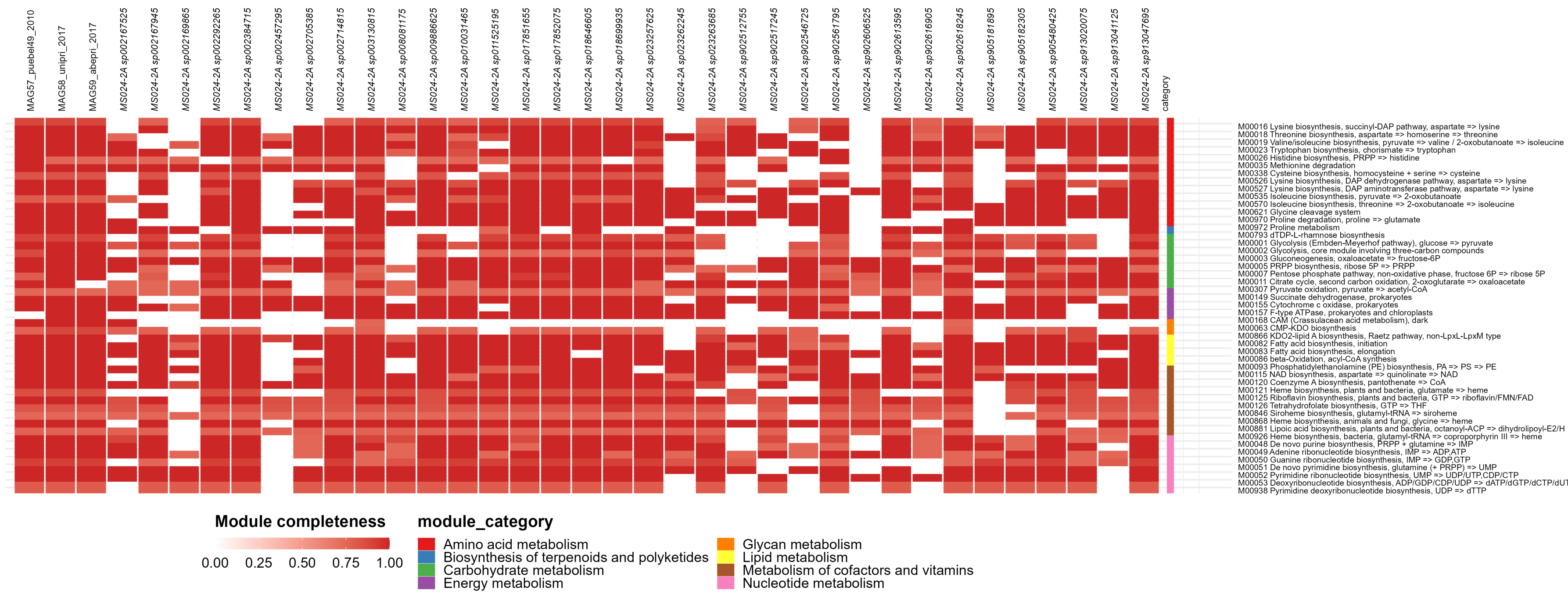


**S26 Fig. Completeness of KEGG metabolic modules in the genus *MS024-2A* (order Flavobacteriales).** Three MAGs (57-59) were recovered in this genus, which relate to free-living seawater species. The color scale represents module completeness, ranging from 0 to 1. Only modules with completeness ≥ 75% in at least one genome are presented.
