# Supplementary material for: Proteobacteria with chemosynthetic potential are highly prevalent in the gills of Hypoplectrus reef fishes
Source: PLoS Genet. 2026 Aug 28;22(8):e1012266. doi: 10.1371/journal.pgen.1012266 (PMC13552952; doi:10.1371/journal.pgen.1012266)
Supplement: Supplementary file 28 [file pgen.1012266.s028.pdf]

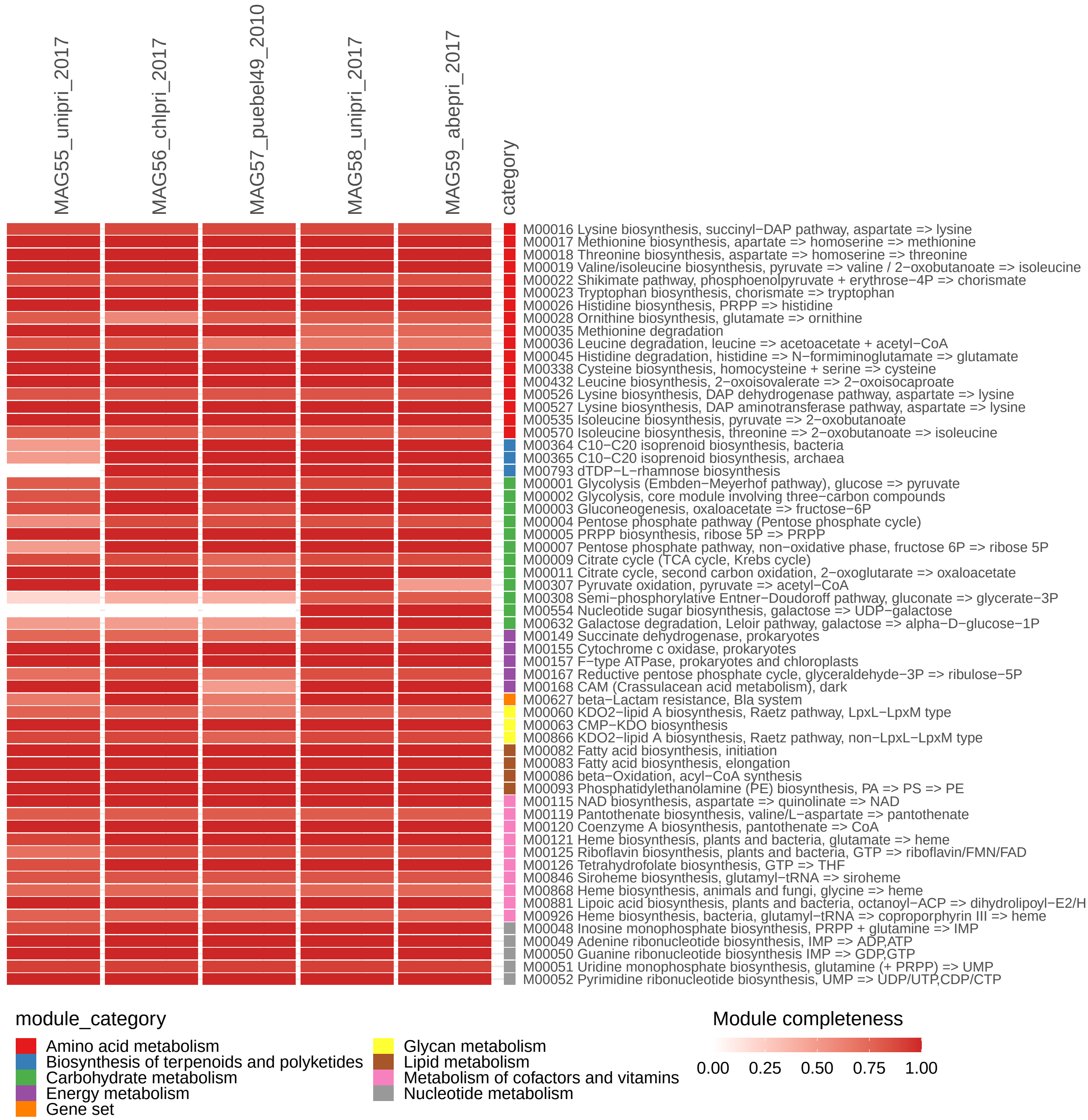

S28 Fig. Completeness of KEGG metabolic modules for the five MAGs in the family Flavobacteriaceae. MAG55-MAG56 belong to genus *Arcticimaribacter* while MAG57-MAG59 belong to the genus *MS024-2A*. The color scale represents the module completeness, ranging from 0 to 1. Only modules with completeness $\geq 75\%$ in at least one genome are presented.
