# Supplementary material for: Proteobacteria with chemosynthetic potential are highly prevalent in the gills of Hypoplectrus reef fishes
Source: PLoS Genet. 2026 Aug 28;22(8):e1012266. doi: 10.1371/journal.pgen.1012266 (PMC13552952; doi:10.1371/journal.pgen.1012266)
Supplement: Supplementary file 29 [file pgen.1012266.s029.pdf]

**Table S1. Marginal PERMANOVA and interaction models for gill microbiome community composition.** PERMANOVA results (adonis2) based on Bray–Curtis dissimilarities calculated from sample-level phylum relative abundance data. The first model presents a marginal PERMANOVA including biogeographic region, host species, sampling location, sampling year, and sequencing run (Seqrun), testing the unique contribution of each factor while controlling for all others. The second and third models test for interactions between fish host species and both sampling year and location, respectively, while accounting for sequencing run and other covariates. The table reports degrees of freedom (Df), sums of squares (SumOfSqs), proportion of variance explained (R2), F-statistic (F) and associated p-values (Pr(¿F)) based on 999 permutations. Because biogeographic region is nested within sampling location, its marginal effect was not estimable in some models.

| **Factor** | **Df** | **SumOfSqs** | $R^2$ | **F** | **Pr(>F)** |
|---|---|---|---|---|---|
| **Marginal PERMANOVA model: Region + Species + Location + Year + Seqrun** | | | | | |
| **Region** | 0 | 0.0000 | 0.0000 | — | — |
| **Species** | 12 | 0.6891 | 0.0256 | 1.6717 | 0.018 |
| **Location** | 4 | 0.1460 | 0.0054 | 1.0626 | 0.381 |
| **Year** | 3 | 0.1352 | 0.0050 | 1.3120 | 0.234 |
| **Seqrun** | 5 | 1.8843 | 0.0700 | 10.9705 | 0.001 |
| **Residual** | 323 | 11.0957 | 0.4124 | — | — |
| **Total** | 352 | 26.9054 | 1.0000 | — | — |
| **Interaction PERMANOVA model: Region + Species * Year + Seqrun** | | | | | |
| **Region** | 2 | 0.1327 | 0.0049 | 1.8909 | 0.092 |
| **Seqrun** | 5 | 1.5745 | 0.0585 | 8.9724 | 0.001 |
| **Species:Year** | 19 | 0.4321 | 0.0161 | 0.6479 | 0.979 |
| **Residual** | 308 | 10.8097 | 0.4018 | — | — |
| **Total** | 352 | 26.9054 | 1.0000 | — | — |
| **Marginal interaction PERMANOVA model: Year + Region + Species * Location + Seqrun** | | | | | |
| **Year** | 3 | 0.1250 | 0.0047 | 1.2089 | 0.277 |
| **Region** | 0 | 0.0000 | 0.0000 | — | — |
| **Seqrun** | 4 | 0.4350 | 0.0162 | 3.1552 | 0.003 |
| **Species:Location** | 25 | 0.8242 | 0.0306 | 0.9565 | 0.586 |
| **Residual** | 298 | 10.2715 | 0.3818 | — | — |
| **Total** | 352 | 26.9054 | 1.0000 | — | — |
