# Supplementary material for: Proteobacteria with chemosynthetic potential are highly prevalent in the gills of Hypoplectrus reef fishes
Source: PLoS Genet. 2026 Aug 28;22(8):e1012266. doi: 10.1371/journal.pgen.1012266 (PMC13552952; doi:10.1371/journal.pgen.1012266)
Supplement: Supplementary file 30 [file pgen.1012266.s030.pdf]

Table S2. Statistical comparison of COG functional category proportions between gill and seawater metagenomes. Mean proportions of each COG category are shown for Bocas 2017 gill and seawater samples. Differences were assessed using two-sample Welch t-tests. P-values were adjusted for multiple testing using the Benjamini–Hochberg false discovery rate (FDR). The column "higher in" indicates the environment with the higher mean proportion.

category	function	mean gill	mean seawater	difference	p-value	adjusted P-value	higher in
C	Energy production and conversion	0.064715	0.093943	-0.029228	1.23e-06	1.54e-05	Seawater
F	Nucleotide metabolism	0.029343	0.054310	-0.024968	7.05e-08	1.76e-06	Seawater
G	Carbohydrate metabolism	0.044979	0.051247	-0.006268	1.55e-03	6.47e-03	Seawater
L	Replication, recombination and repair	0.071434	0.092245	-0.020811	6.87e-03	2.45e-02	Seawater
N	Cell motility	0.014263	0.006728	0.007535	2.07e-06	1.72e-05	Gill
V	Defense mechanisms	0.045147	0.021531	0.023616	7.48e-04	3.74e-03	Gill
X	Mobilome (phages, transposons)	0.055969	0.029750	0.026219	1.37e-04	8.57e-04	Gill
